# Supplementary material for: Physical deep learning with biologically inspired training method: gradient-free approach for physical hardware
Source: Nat Commun. 2022 Dec 26;13:7847. doi: 10.1038/s41467-022-35216-2 (PMC9792515; doi:10.1038/s41467-022-35216-2)
Supplement: Supplementary file 1 — Supplementary Information [file 41467_2022_35216_MOESM1_ESM.pdf]

Supplementary Information for Physical Deep Learning with Biologically Inspired Training Method: Gradient-Free Approach for Physical Hardware

Mitsumasa Nakajima[1], Katsuma Inoue[2], Kenji Tanaka[1], Yasuo Kuniyoshi[2,3], Toshikazu Hashimoto[1], and Kohei Nakajima[2,3]

1. *NTT Device Technology Labs., 3-1 Morinosato-Wakamiya, Atsugi, Kanagwa, Japan*
2. *Graduate School of Information Science and Technology, The University of Tokyo*
3. *Next Generation Artificial Intelligence Research Center, The University of Tokyo*

Supplementary note S1. Investigation towards application of DFA to broader network models

Current physical implementations of neural networks are limited to a simple model such as RC and MLP. We demonstrated the applicability of the augmented DFA to these models through the simulations and physical experiments described in the main article. Here, we consider the scalability of the DFA-based approach to more modern models. One of the most commonly used models for the standard deep learning is a deeply connected convolutional neural network (CNNs such as AlexNet and VGG. However, it was reported that the DFA algorithm is difficult to apply to CNNs[1,2].Thus, the proposed method may be difficult to apply to convolutional PNNs[3–5] in a simple manner.

On the other hand, a recent study revealed that a full-connection network alone, named MLP-Mixer, can achieve state-of-the-art performance[6]. Although the DFA-based training may be effective for such convolution-free models, there have been no reports on the applicability of DFA for MLP-Mixer[6]. In addition, it has also been reported that DFA can train modern network architectures, including a graph neural network and transformer, without a convolution layer [7]. Those results suggest that our algorithm might work on such practical network structures. Considering analog hardware implementations, the applicability to spiking neural networks (SNNs) is also an important topic[8,9]. The suitability of DFA-based training for SNNs has been reported[10], which suggests that our proposed augmented DFA could make the training easier. Regarding DFA for the CNN-based model, an investigation in a previous study was limited to models without skip connections. It was reported that the DFA angle increases with depth, which leads to failure of the training[1]. At the same time, it has been reported that the alignment angle in the convolution layer near the final layer is small enough even in the CNN, suggesting a shallow path to the final layer is one key to the success of DFA-based training even in the CNN. Notably, it has been reported that forming skip connections is equivalent to

forming an ensemble of deep and shallow networks[11]. In addition, it was also reported that most of the effective gradients in ResNet came from the shallow path. Thus, it is expected that ensembled shallow paths would have a positive impact on DFA-based training. In such a network, there remains the possibility of successful DFA training even for deep CNNs.

While the DFA-based algorithm has potential to scale to the above-mentioned more practical models beyond the simple MLP or RC, the application of DFA-based training to such networks has not been investigated yet. Here, we examined the scalability of DFA-based training (DFA itself and augmented DFA) to the above models (MLP-Mixer, Vision transformer, SNNs, and ResNet). We found that the DFA-based training is effective even for the explored practical models. While the achievable accuracy of DFA-based training is basically lower than BP training, some tuning of the model and algorithm could improve the performance. Notably, the accuracy of DFA and augmented DFA is almost comparable for all the explored experimental setups, suggesting that further improvement of the DFA itself directly contributes the improvement of the augmented DFA. For the experiments in this section, we utilized a Pytorch-based DFA module that is available in github[12].

Supplementary note S1.1. MLP-mixer

Here, we investigated the application of DFA-based training to the MLP-Mixer[6]. Although the MLP-Mixer is basically composed of fully connected (FC) layers, it differs from a standard MLP model in the following points: (i) MLP-Mixer has a patch embedding layer; (ii) the patch-embedded inputs typically have three-dimensional data [dim(batch, channels, patches)], which are treated as time-series data; and (iii) the outputs from each MLP layer are transposed to take the connection in both directions on the channel and patch axes. As a starting point, we investigated whether these unique treatments are acceptable for DFA-based training.

First, we investigated the effect of the time-series processing in MLP-Mixer using the experimental setup shown in Fig. S1(a). In this experiment, MNIST images treated as time-series. In this structure, we can ignore the influence of patch embedding and the transposition processing in the MLP-mixer, enabling the investigation on the time-series treatment itself. We employed four FC layers with 100 nodes. The Relu function was used for the nonlinear activation, and Adam with a learning rate of 1e-3 was used for the optimizer. Before the final layer, we introduced a downsampling layer by averaging in the time direction like in MLP-Mixer. As an output of the forward path, we obtained 1×10 (number of classes) final error. In the DFA training path, we upconverted the final error from 1×10 to 28 (image size) ×10. For the upconversion, we simply copied the error along time direction. The upconverted signals were weighted by a random matrix with the size of 10 ×100 (number of nodes in FC layers), the same as in the standard DFA approach. We used the same random matrix along time direction. To investigate the adaptivity of our proposed augmented DFA, we also examined the case for g=sin(a) instead of f'(a)=1/cosh^2(a). Figure S1(c) shows the evolution for the BP, DFA and augmented DFA

training. As a baseline, we also plotted the results for a network whose parameters were fixed without the final layer (labeled as "final-layer-only" in the figure). Note that the achievable accuracy was inferior to standard MLP even in the BP case because the examined architecture does not have a connection on the time axis. As can be seen in the figure, the DFA-based training is clearly superior to final-layer-only training, while the accuracy is slightly inferior to the BP. These results suggest the both DFA and augmented DFA can be applied to the input data with time-series treatment.

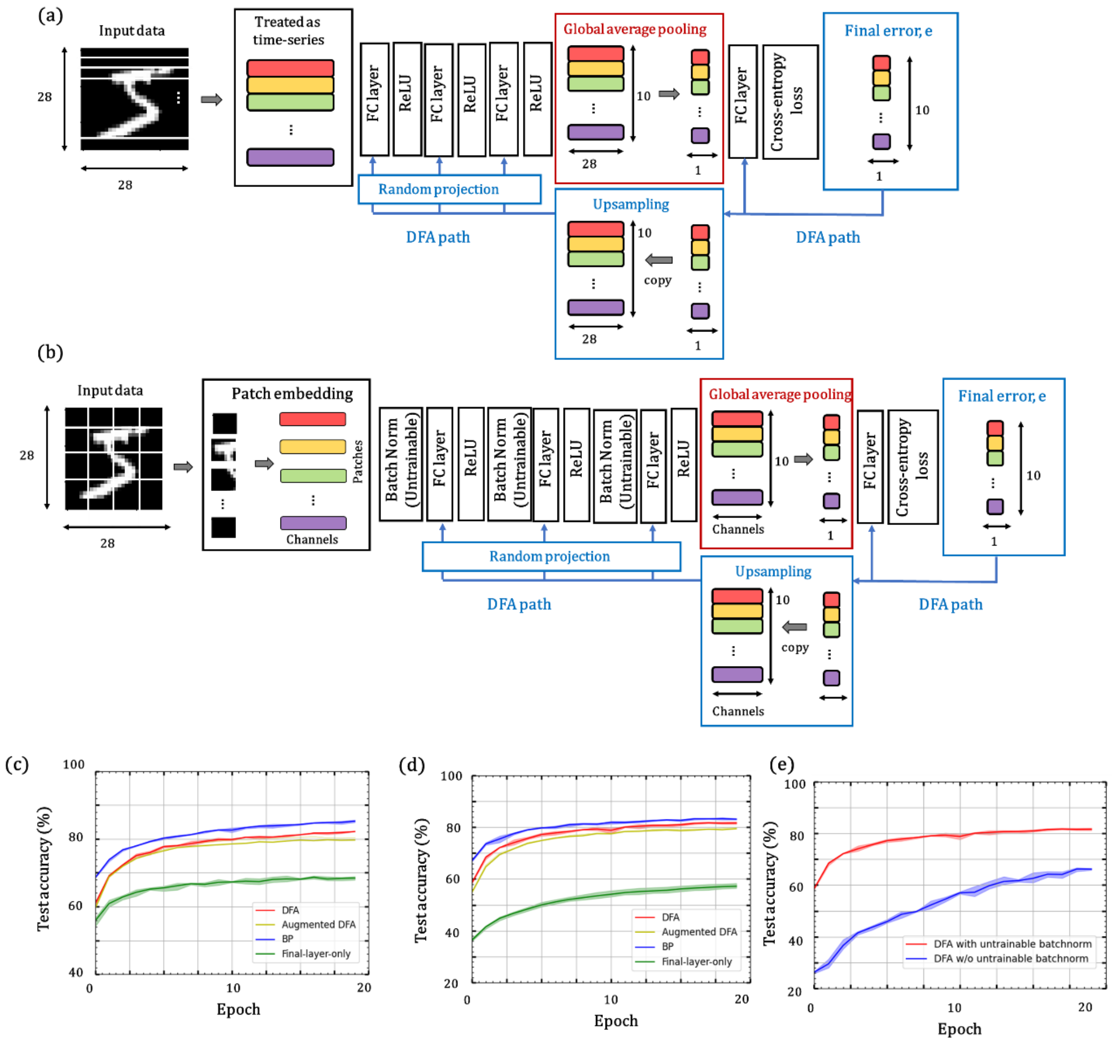

Fig. S1. ***Augmented DFA for MLP-mixer-based network.*** *Schematic of experimental setup for (a) time-series treatment and (b) patch embedding with DFA-based training. Evolution of test accuracy for the experiment with (c) time-series treatment and (d) patch embedding. Red, yellow, blue, and green solid lines show the results with DFA, augmented DFA, BP and final-layer-only training. (e) Test accuracy with (red) and without (blue) untrainable batch normalization. Data in this figure were obtained using standard CPU/GPU computation. Each experiment was repeated five times.*

Next, we investigated the effect of patch embedding in MLP-Mixer. The experimental setup is shown in Fig. S1(b). In this architecture, we added a patch embedding layer on the model shown in

Fig. S1(a). Unfortunately, adding the patch embedding layer drastically degrades the speed of convergence. Then we added batch normalization (BN) without the trainable affine transformation (untrainable BN) after each FC layer [e.g. we can implement it as *batchnorm1d(affine=False)* for the Pytorch case]. As the untrainable BN layer does not include trainable parameters, it does not affect to the complexity of DFA and augmented DFA training. The results for MNIST training for the DFA, augmented DFA, BP, and final-layer-only are shown in Fig. S1(d). The results for the DFA training with and without untrainable BN are also plotted in Fig. S1(e). As can be seen in the Fig. S1(e), adding the untrainable BN layer clearly accelerates the convergence of the training like standard BN. While the previous study implied that the BN was not effective for DFA training[1], this result suggest that the untrainable BN is still effective for the faster convergence. As can be seen in Fig. S1(d), the difference in the accuracy between the DFA-based and BP training was small, and these accuracies are clearly superior to final-layer-only training. These results suggest that, with the untrainable BN layer added, DFA-based training is still effective for the model with the patch embedding layer.

Next, we constructed MLP-Mixer with the transposition included. The experimental setup is shown in Fig. S2(a), which is based on the standard three-mixer-layer (MLP-Mixer3). The basic structure is the same as that in the original MLP-mixer study[6] except we used untrainable layer normalization (LN) and nonlinear activation of ReLU instead of the original GeLU function. For the augmented DFA training, we used alternative nonlinear function g=sin(a) instead of accurate differential $f'(a)=1/\cosh^2(a)$. Figure S3(a) and (b) show the results for the DFA training (purple line) and BP (blue line) on MNIST [Fig. S3(a)] and CIFAR10 [Fig. S3(b)] benchmarks. For comparison, we also plotted the performance for the final-layer-only training (green line). As shown in the figure, the performance of the model with DFA training is inferior to that of BP, especially regarding the accuracy for the CIFAR10, which is almost the same as that for the final-layer-only training. This suggests that introducing the transposition causes the DFA training to fail. This performance degradation could be due to the difference in the direction for the transposed matrix and error matrix, as shown in the inset of Fig. S2(a). In MLP-Mixer, the outputs from the mixer layers are averaged along the channel direction. Thus, the final error only includes the information along the patch axis, which might result in the failure of the transposed MLP layer [the first MLP in the Fig. S2(a)]. Here, we tried a new experimental setup as shown in Fig. S2(b). In this setup, we used dual FC layers; one treats the signals by transposing the inputs, and the other treats them without transposition. After the dual FC layer, the signals are summed, and the sum contains both transposed and non-transposed information. By feeding the error signals from this output layer back, we can train both the transposed and non-transposed layer using the DFA algorithm. We refer to this method as dual-final-layer (DFL). The results for the DFA training for the model with DFL are also plotted in Fig. S3(a) and (b) as yellow solid lines. As can be seen in the figure, introducing DFL increased the accuracy, suggesting the success of the training even in the transposed layer. In particular, for MNIST in Fig. S3(a), the accuracy

was almost same as with BP. These results suggest that DFA and augmented DFA are applicable to the MLP-Mixer-based model. Unfortunately, for CIFAR10 in Fig. S3(b), the accuracy achievable with DFA-based training was still lower than that for BP training, suggesting that further improvement of the DFA algorithm or target model is required for larger scale datasets. On the other hand, the accuracies of the DFA and augmented DFA training are comparable for both the MNIST and CIFAR10 benchmarks. This suggests that the performance of the augmented DFA is limited by the the performance of DFA itself. Thus, by improving the DFA algorithm itself (such as through our DFL approach), we could achieve more competitive performance even in the gradient-free augmented DFA algorithm.

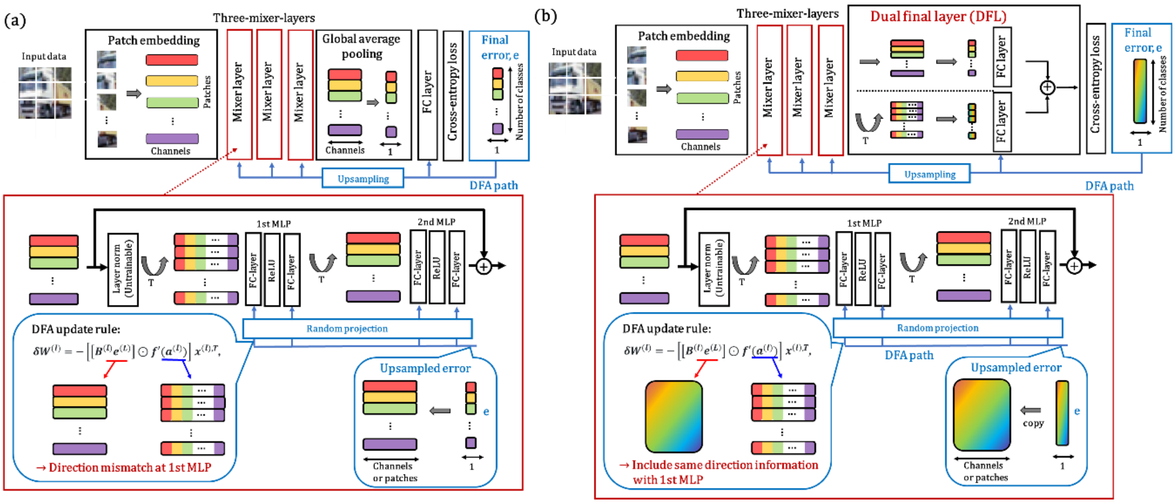

Fig. S2. ***Custom MLP-mixer for augmented DFA.*** *Schematic of experimental setup for (a) standard and (b) improved MLP-Mixer with three mixer layers with DFA training. The improved MLP-Mixer has dual final layers (DFLs) which include the same directional information as both the first and second MLP.*

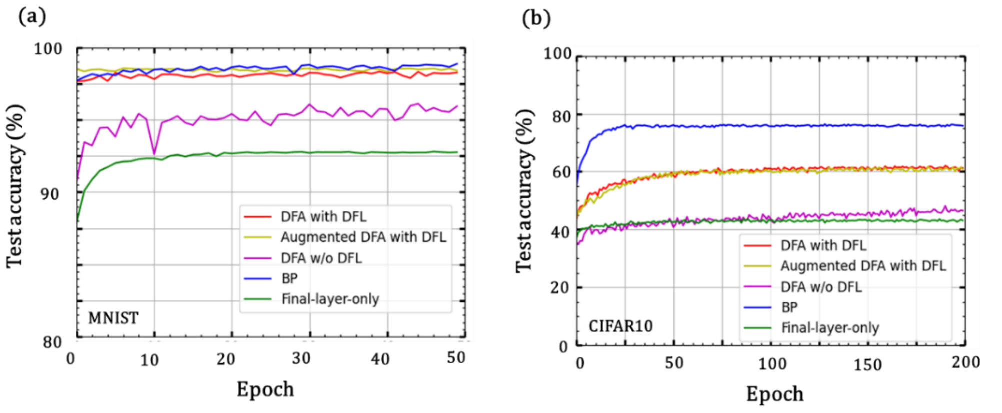

Fig. S3. ***Performance of MLP-mixer with augmented DFA training.*** *Evolution of test accuracy for the experiment for (a) MNIST and (d) CIFAR10 datasets. Red, yellow, purple, blue, and green solid lines show the results with DFA using DFL, augmented DFA using DFL, DFA without DFL, BP, and final-layer-only training. Data in this figure were obtained using standard CPU/GPU computation.*

Supplementary note S1.2 ResNet

Next, we investigated application of DFA to convolutional neural networks (CNNs) with skip connections, which is called ResNet[13]. Although it has been shown theoretically and experimentally that it is difficult to train deep convolutional layers using DFA, it has been reported that the convolution layer near the final layer can be trained even in the CNN, suggesting a shallow path to the final layer is key to the success of the DFA-based training in a CNN. Here, we focused on ResNet because it has been reported that forming skip connections in ResNet is equivalent to forming an ensemble of deep and shallow networks [see Fig. S4(b)]. Thus, it is expected that ensembled shallow paths will have a positive impact on DFA training. In such a network, there remains the possibility of successful DFA training, even for deep CNNs.

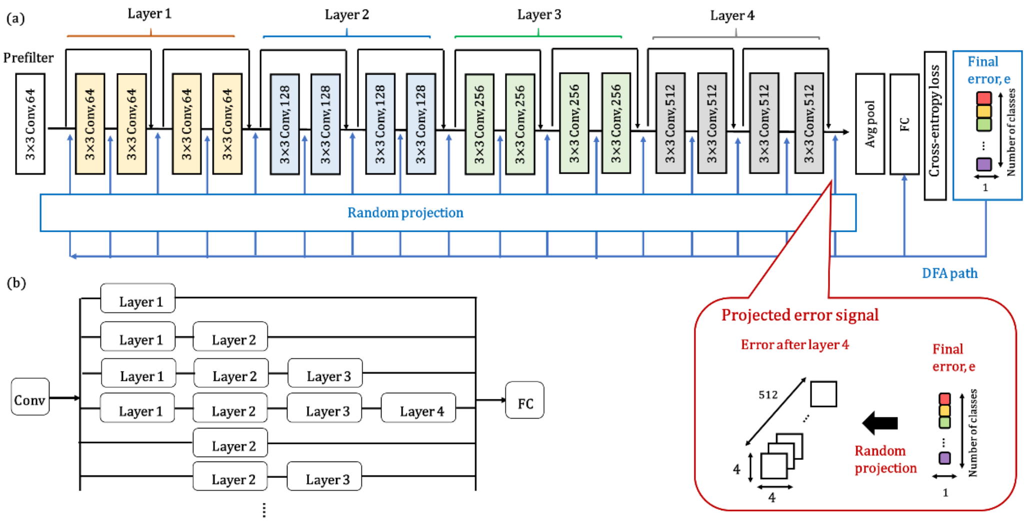

*Fig. S4. **Augmented DFA for ResNet.** (a) Schematic of experimental setup for ResNet with DFA-based training. (b) Equilibrium structure of ResNet, which can be considered as ensemble of shallow and deep networks.*

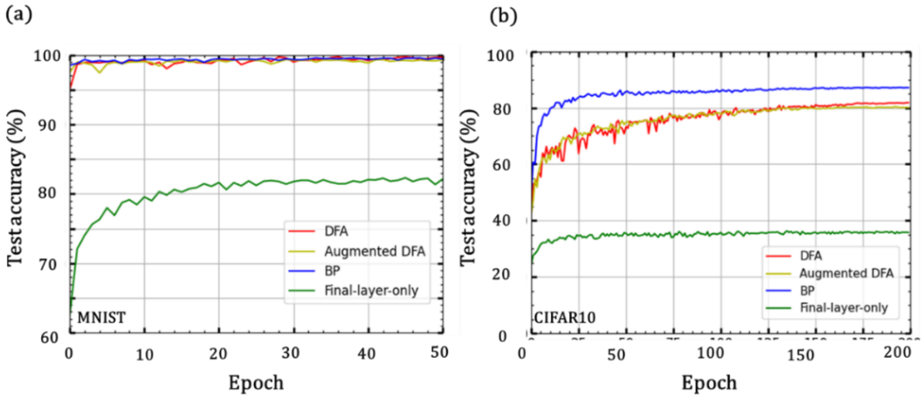

*Fig. S5. **Performance of ResNet with augmented DFA training.** Evolution of test accuracy for (a) MNIST and (d) CIFAR10 datasets. Red, yellow, blue, and green solid lines show the results with DFA, augmented DFA, BP, and final-layer-only training. Data in this figure were obtained using standard CPU/GPU computation. Each experiment was repeated five times.*

Here, we applied DFA-based training to ResNet. Figure S4(a) shows the experimental setup, which is based on ResNet-18. The operation of the forward path is almost same as in the standard ResNet18, except that we added an untrainable BN layer after each convolution layer. Note that the untrainable BN layers are not illustrated in the figure for simplicity. Also, we used only 3×3 convolution as the first downsampling layer instead of the original 7×7 and 3×3 convolution layers so that we could adopt compact MNIST and CIFAR10 datasets. Thus, the total number of convolution layer is 17 (not 18). As an output of forward propagation, we obtained 1×10 (number of classes) final error signal. In the DFA training path, the final error was randomly converted by the random matrix so that it would have the same shape as the outputs in each layer [e.g. 512 (filter number) × 4 × 4 (image shape) at layer 4]. As benchmark datasets, we used MNIST and CIFAR-10. Adam with a learning rate of 1e-4 was used for the optimizer. For the augmented DFA training, we used alternative nonlinear function g=sin(a) instead of the accurate differential $f'(a)=1/\cosh^2(a)$.

Figure S5(a) and (b) show the results for the training with DFA (purple line) and BP (blue line) using the MNIST [Fig. S5(a)] and CIFAR10 [Fig. S5(b)] benchmarks. For comparison, the performance for final-layer-only training (green line) is also plotted. As shown in the figures, the performance of the model with DFA is far superior to that for final-layer-only training, suggesting the success of the training. In particular, the accuracies for the DFA and augmented DFA training were almost the same that for the BP training in the case of the MNIST dataset. Although the accuracies of the DFA-based training for the CIFAR10 dataset were still relatively lower than that for the BP training, the obtained moderate accuracies support that the DFA-based training is effective even in a convolution-based architecture with skip-connection paths.

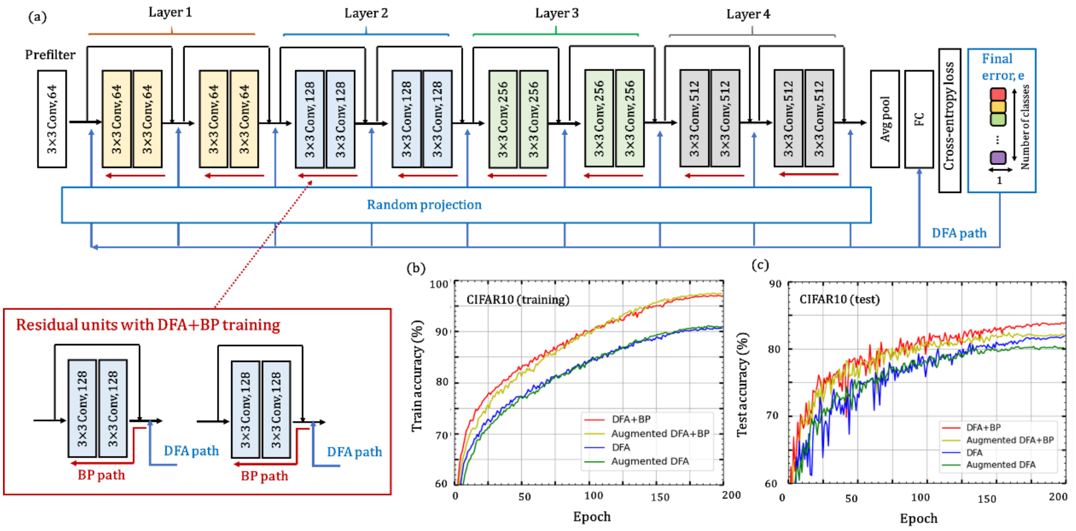

Fig. S6. ***Hybrid Training for ResNet.*** *(a) Schematic of experimental setup for ResNet with DFA+BP training. Evolution of (b)training and (c) test accuracy for CIFAR10 dataset. Red, yellow, blue, and green solid lines show the results with DFA+BP, augmented DFA+BP, BP, and final-layer-only training. Data in this figure were obtained using standard CPU/GPU computation.*

To explore a method for achieving higher accuracy, we also examined the combination of BP and DFA training shown in Fig. S6(a). In this architecture, the final error signals are fed back after each residual block instead of after each convolution layer, which is almost same manner in the method called macro-DFA in the work by Launay *et al* [12]. In each block, we used the BP to train the first convolution layer. Although we used the BP partially, the training is still based on the parallel random projection. Thus, the advantages of the DFA and augmented DFA are still effective. Figure S6(b) shows the accuracy for the combination of DFA and BP. The accuracy was improved by using the BP+DFA combination scheme [training accuracy from 90.68 to 97.06% (DFA) and from 91.14 to 97.45% (augmented DFA); test accuracy from 81.69 to 83.86% (DFA) and from 80.38 to 82.16% (augmented DFA)]. The results suggest that there is still room for improving performance by tuning or combining the algorithms.

Supplementary note S1.3 Vision transformer

Next, we examined the applicability of DFA training to Vision Transformer (ViT)[14]. Note that the application of the DFA training to the transformer-based models for natural language processing (NLP) have already been demonstrated by Launay *et al* [12]. Figure S7(a) shows the experimental setup for the ViT model, which is based on the standard three-layer ViT (ViT-3). The ViT comprises patch embedding layer and multiple transformer layers. Each transformer layer includes multi-head attention and MLP with an LN layer. Unlike the standard ViT, we used an untrainable LN layer for the DFA-based training. The Relu function was used for the nonlinear activation, and Adam with a learning rate of 1e-4 was used for the optimizer. Before the final layer, a downsampling layer was introduced for averaging the data along the channel direction. As an output of the forward path, we obtained 1×10 (number of classes) final error. In the DFA training path, we upconverted the final error to (channel size) × (number of classes). For the upconversion, we simply copied the error along the channel axis. The upconverted signals were weighted by the random matrix, the same as in the standard DFA approach. In this study, we only examined the combination of BP and DFA described in section 11.2; each error signal was fed back after each transformer block. For the augmented DFA training, we used alternative nonlinear function $g(a)=\sin(a)$ instead of the accurate differential $f'(a)=1/\cosh^2(a)$.

The results for the MNIST and CIFAR10 benchmarks are shown in Fig. S7(b) and (c). For comparison, the performance for the final-layer-only training is also plotted. Note that the relatively low performance even for BP is due to the lack of fine-tuning (ViT-based architectures typically require large-scale fine tuning to achieve competitive performance). As can be seen in the figure, the trends are almost same as with the other model (MLP-Mixer and ResNet) case; the accuracies of the DFA and augmented DFA were almost the same as that of BP for MNIST, but they were relatively lower than BP for CIFAR10. This suggests that the DFA and augmented DFA training is effective for transformer-based models. The accuracies of the DFA and augmented DFA training were almost

comparable for both the MNIST and CIFAR10 benchmarks, as they were for the other models. This suggest that the performance of augmented DFA is limited by the performance of DFA itself, which might be improved through further investigation of DFA-based training in future.

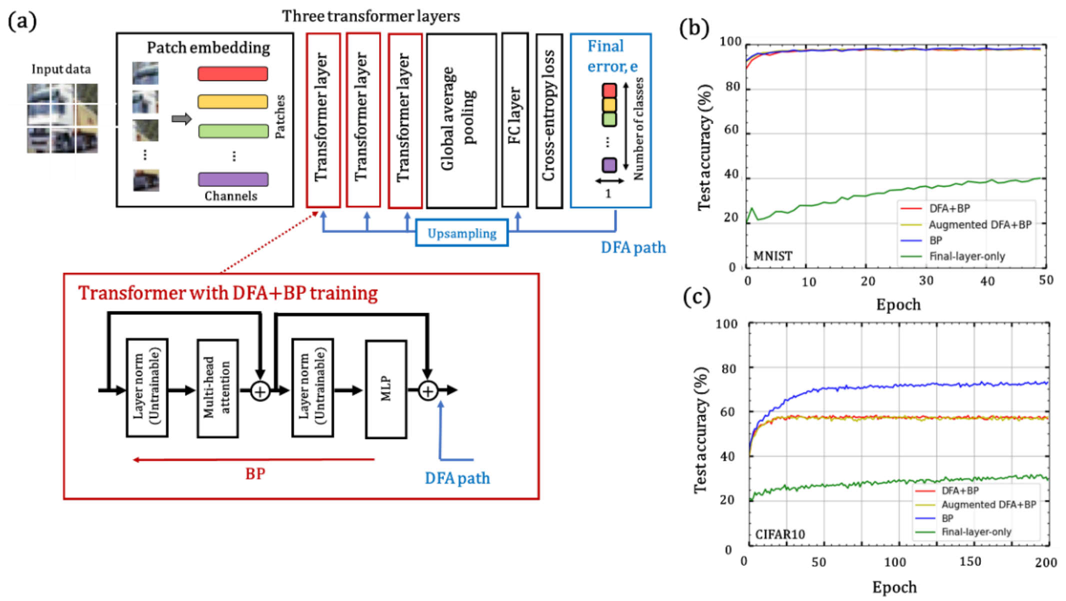

Fig. S7. ***Augmented DFA for ViT.*** *(a) Schematic of experimental setup for ViT with DFA+BP training. Evolution of test accuracy for (b) MNIST and (c) CIFAR10 datasets. Red, yellow, blue, and green solid line show the results with DFA+BP, augmented DFA+BP, BP, and final-layer-only training. Data in this figure were obtained using standard CPU/GPU computation. Each experiment was repeated five times.*

Supplementary note S1.4 Spiking neural networks

Next, we examined the effectiveness of our augmented DFA algorithm in spiking neural networks (SNNs), a type of artificial neural network that mimics natural neural networks more closely. Our gradient-free approach would be particularly useful in SNNs since it is generally difficult to obtain the gradient information about spiking neurons owing to the discrete manner of spike transmission. Samadi *et al.* [10] have recently shown that a DFA-like algorithm called broadcast alignment (BA) successfully learns MNIST on SNNs, in which they introduced an approximated derivative function of average spiking activities obtained by their empirical observations. They also introduced a derivative-free version, equivalent to using the identity function as g in the augmented DFA, and found that it fails to learn nearly as well as BA, from which they claimed that it is useful to reflect the properties of the feedforward process to its direct backward pathway.

Here, we went a step further by applying our augmented DFA to SNNs; that is, we investigated the applicability of the augmented DFA approach to the SNN model by replacing the approximated derivative function with nonlinear functions physically available in our optical setups.

```
set Δt = 0.25       // time step
set τ = 2           // time constant
set h_th = 0.4      // threshold
set ε = 10**-8      // tiny positive number

// see the supplementary material in the original paper for initialization processes
initialize(W)       // feedforward weights
initialize(b)       // biases
initialize(W)       // feedback weights
initialize(v)       // drives
initialize(h)       // hillock potentials
initialize(a)       // activities
initialize(ref)     // times since refractory periods began
initialize(μ)       // d learning rate constants η

foreach ((x, d) in datasets) do
    a[0] = x
    set t = 0
    while(t < 100) do
        // feedforward process
        for (set n = 1; n <= num_layer; n++) do     // for each layer
            set v[n] = W[n].dot(a[n - 1]) + b[i]
            set ref[n] = (ref[n] > 0) * Δt           // how long has the cell been ref?
            set ref[n] = (ref[n] <= 1) * ref[n]      // end refractory period
            set h[n] = (ref[n] > 0) * (h[n] + (v[n] - h[n]) * (Δt / τ))
            set ref[n] = (h[n] < h_th) * ε + (h[n] >= h_th) * ref[n]
            set a[n] = (ref[n] > 0)
        end
        // feedback process
        if (t > 20) do
            set e = a[num_layer] - d
            for (set n = num_layer; n >= 2; n--) do
                set δ[n] = B[n].dot(e)              // direct path
                set ι[n] = μ[n] * δ[n] * g(v[n])    // a-DFA
                set W[n] = W[n] - ι[n].dot(a[n - 1])
                set b[n] = b[n] - ι[n]
            end
        end
        t += Δt
    end
end
```

Fig. S8 ***Pseudo-code for SNN with DFA training.*** *Pseudo-code for SNN with augmented DFA training is displayed. Although the set up is almost same with previous study* [10]*, we extended the nonlinearity to alternative function g.*

The experimental setup was the same as the one for BA, i.e., we prepared the same three-layer SNN (784-1000-10) for MNIST with leaky-integrate-and-fire neurons optimized by the training process shown in Fig. S8. For our augmented DFA setup, the gradient was set by optical function g(a) = $\cos^2(\omega a + \theta)$, the amplitude ω and phase θ of which were swept through {0.05, 0.10} and {0°, 15°, 30°, 45°, 60°, 75°, 90°, 105°, 120°, 135°, 150°, 165°, 180°} respectively, generating nonlinear functions with various η (correlation coefficient between g and f'). Fig. S9 depicts the test accuracies as a function of θ. For comparison, results for the BA with the approximated derivative function ($g(a) = \cdots$), DFA and BP with actual derivative function ($g(a) = f'(a) = \cdots$), and derivative free version ($g(a) = 1$) are overwritten as horizontal lines. The results show that augmented DFA obtains

moderate performance of 98%, which is competitive not only with BA but also with BP and DFA using actual *f'* especially around θ=150°~165°, where *η* [i.e. corr(f', g)] gets relatively high. In addition, our augmented DFA exhibit robustness against the θ value, suggesting a wide range of improvised cos-based functions can be substituted for the nonlinearity required in the DFA-based training. These results show that our gradient-free approach is valid even in SNN models, supporting the scalability and effectiveness of the augmented DFA.

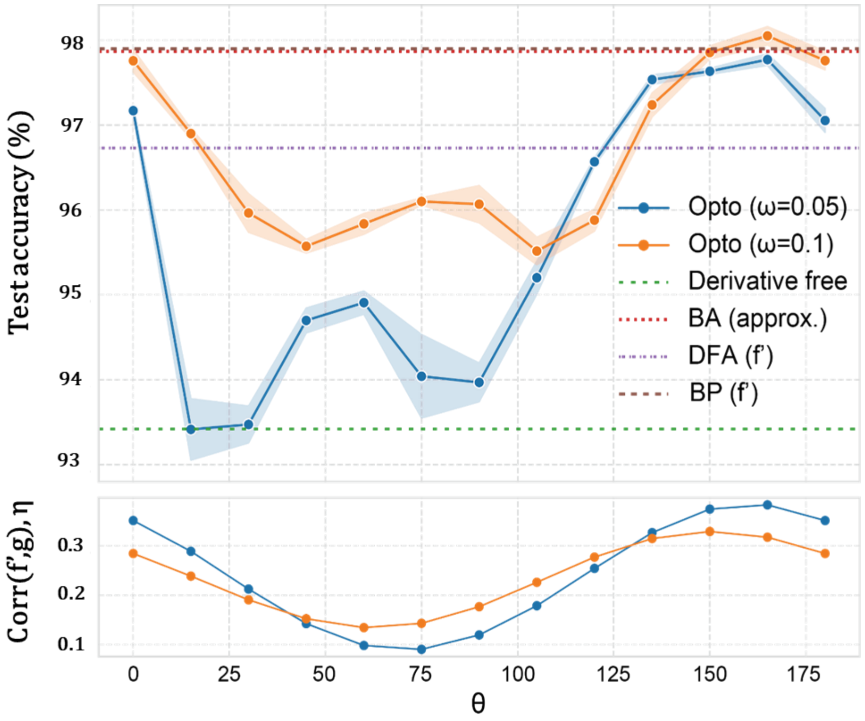

Fig. S9. ***Performance of SNN with augmented DFA training.*** *Applicability of the augmented DFA toSNNs. [Top] Test accuracy as a function of angle θ in* $g(a) = cos^2(\omega a + \theta)$. *The four horizontal lines correspond to accuracies with derivative-free, BA, DFA, and BP models, respectively. Average performance over five trials is displayed for the augmented DFA, derivative-free, and BA models. On the other hand, the best scores among the five trials are shown for DFA and BP models since the training became unstable owing to the singular point in f'. [Bottom] Correlation coefficient η between g and f'. For stability of calculation, f' was clipped in the range of [0, 1].*

Supplementary note S2. Application to another photonic system

Unitary nanophotonic deep neural network As an example of augmented DFA for a physical neural network, we show a numerical simulation of a unitary neural network composed of a nanophotonic unitary processor[15,16]. As shown in Fig. S10(a), the nanophotonic processor is composed of arrays of 2 × 2 unitary operator R_{ij} based on a Mach-Zehnder interferometer (MZI). Any $N \times N$ unitary matrix U can be decomposed as a product of R_{ij} and diagonal matrix D, such that $U = D \prod_{i=2}^{N} \prod_{j=1}^{i-1} R_{ij}$, where R_{ij} is defined as an N-dimensional identity matrix with the elements R_{ii}, R_{ij} , R_{ji}, and R_{jj} replaced as follows:

$$\begin{pmatrix} R_{ii} & R_{ij} \\ R_{ji} & R_{jj} \end{pmatrix} = \begin{pmatrix} e^{i\varphi}cos\theta & -e^{i\varphi}sin\theta \\ sin\theta & cos\theta \end{pmatrix}, \tag{S1}$$

where φ and θ are the phases in the 2 × 2 unitary operator, which correspond to the phases in the MZI. Therefore, the MZI array can perform arbitral unitary operation. By adding nonlinear activation *f(a)*, the unitary neural network, described as $x^{(l+1)} = f\left(U^{(l)}x^{(l)}\right)$, can be constructed by using the optical system shown in Fig. S10(a), where $U^{(l)}$ is the unitary weight matrix and $x^{(l)} \in \mathbb{C}$ is the complex-valued input/output electric fields. This arrangement is energy-efficient because the unitary operation does not require gain and loss of input light. The gradient of unitary matrix $\delta U^{(l)}$ can be described by

$$\delta U^{(l)} = \frac{\partial E}{\partial U^{(l)}} = -x^{(l),T}\{\left[B^{(l)}e^{(L)}\right] \odot g(a^{(l)})\}, \tag{S2}$$

As the considered device is composed of a unitary matrix operator and trainable parameters, random projection matrix *B* is sampled from complex-value. From eq. (S2), the gradient of phases in the MZI ($\delta\theta^{(l)}$ and $\delta\varphi^{(l)}$) can be obtained from the relationships $\delta\theta^{(l)}=(\partial U^{(l)}/\partial\theta^{(l)})\delta U^{(l)}$ and $\delta\varphi^{(l)}=(\partial U^{(l)}/\partial\varphi^{(l)})\delta U^{(l)}$, where gradient ($\partial U^{(l)}/\partial\varphi^{(l)}$) and ($\partial U^{(l)}/\partial\theta^{(l)}$) can be solved by the adjoint sensitivity method. Therefore, we can obtain $\delta\theta$ and $\delta\varphi$ using the random projection of $e^{(L)}$, the phase gradient against *U* ($\partial U/\partial\theta$ and $\partial U/\partial\varphi$), and arbitrary nonlinear projection *g(a)*. In addition, by introducing random unitary weight $B^{(l)}_{unitary}$ as a random projection matrix in eq. (S2), we can compute $\delta U^{(l)}$ by using an optical system with almost the same configuration as that for forward propagation [see Fig. S10(b)]. In this system, we can execute unitary random projection of $e^{(L)}$ using a photonic unitary processor by setting the random MZI phases. The Hadamard products with *g(a)* are executed by using a beam splitter and optoelectric modulators based on an MZI whose nonlinear activation is described as $g(a) = \sin(a+\Phi_{bias})$, where Φ_{bias} is the control bias of the MZI. As described in the main text, we should avoid the point near $\eta = 0$ [i.e. *g(a)* is near the orthogonal function of $f'(a)$]. By scanning Φ_{bias}, we can avoid this condition. The computational cost of eq. (S2) is O (N^2L). Therefore, there are advantages of optical implementation in computation speed and energy-consumption, the same as in forward propagation. Note that we need to solve adjoint sensitivity method for this application, which requires the knowledge of physical system. Thus, one important feature of augmented DFA (training without pre-knowledge of the physical system) is ineffective at this time. Further improvement of algorithm is future work.

As a demonstration, we executed the numerical simulation for the MNIST task considering the optical setup shown in Fig. S10(a) and (b). We assume an *L*-layered unitary neural network with 64

nodes, where L is scanned from 1 to 10. Thus, each network is composed of a 64 × 64 MZI array. For the nonlinear function, we selected the optoelectric nonlinearity $f(a)$=tanh($|a^{(l)}|^2$) assuming the intensity detection and electrical tanh activation[17]. Since the differential of tanh(a) is even function [{tanh(a)}'=1/cosh^2(a)], we use odd function $g(a)$=cos(a) to avoid the point near η = 0. This condition can be realized by setting Φ_{bias}=π/2. As the MZI has only 64 input ports, the input images were reshaped into an 8 × 8 size, and they were arranged to a 64-sized one-directional vector. The layer dependency of test error for the MNIST task is shown in Fig. S10(c). For comparison, we also plot the results using the BP training with $f'(a)$ replaced with $g(a)$. As can be seen in the figure, we could successfully train the nanophotonic unitary neural network even using augmented DFA. The results also suggest that the DFA approach is effective for complex-valued unitary neural networks. The accuracy for the BP training became worse when the network became deeper because $f'(a)$ was replaced to $g(a)$, which suggests that augmented DFA is more effective for deep networks. Although the achievable accuracies for relatively shallow networks were inferior to those for BP, the augmented DFA is still considered useful because we do not need to solve BP and eq. (S2) can be solved with an optical processor.

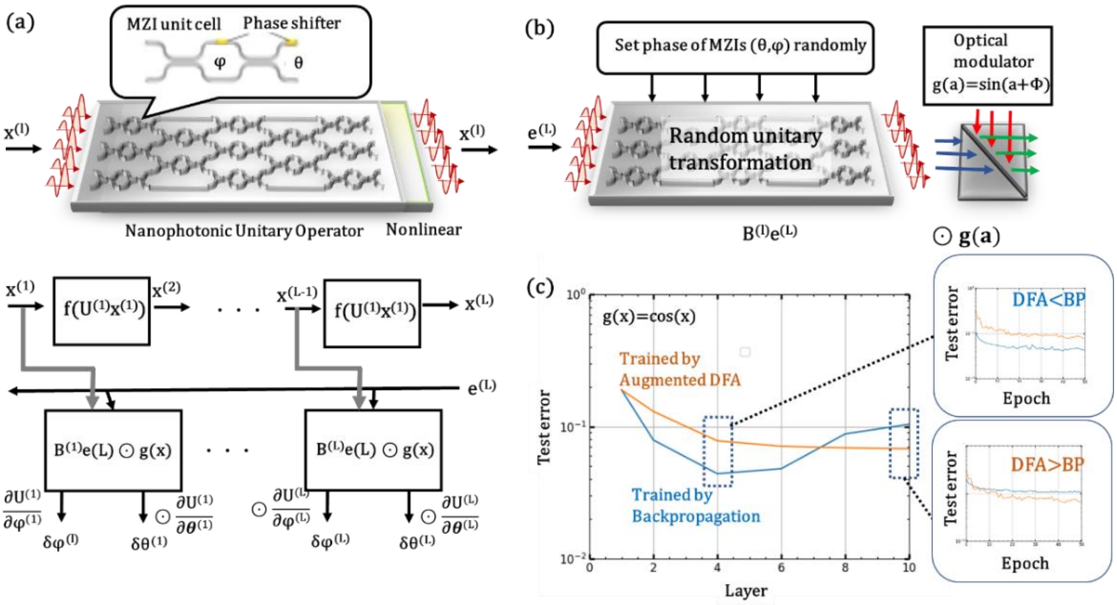

Fig. S10. ***Augmented DFA for nanophotonic unitary neural network.*** *(a) Schematic illustration of deep unitary neural network composed of unitary nanophotonic operator. (b) Implementation of optical solver for random projection. (c) Numerical experimental test error as a function of the number of the network layer. Data in this figure were obtained using standard CPU/GPU computation.*

Diffractive deep neural networks As another demonstration of DFA in a photonic system, we show the inverse optimization of a diffractive deep neural network (D^{2}NN). Figure S.11(a) shows the basic operation of the D^{2}NN[18–20]. The D^{2}NN is composed of diffractive optical elements (DOEs), which can

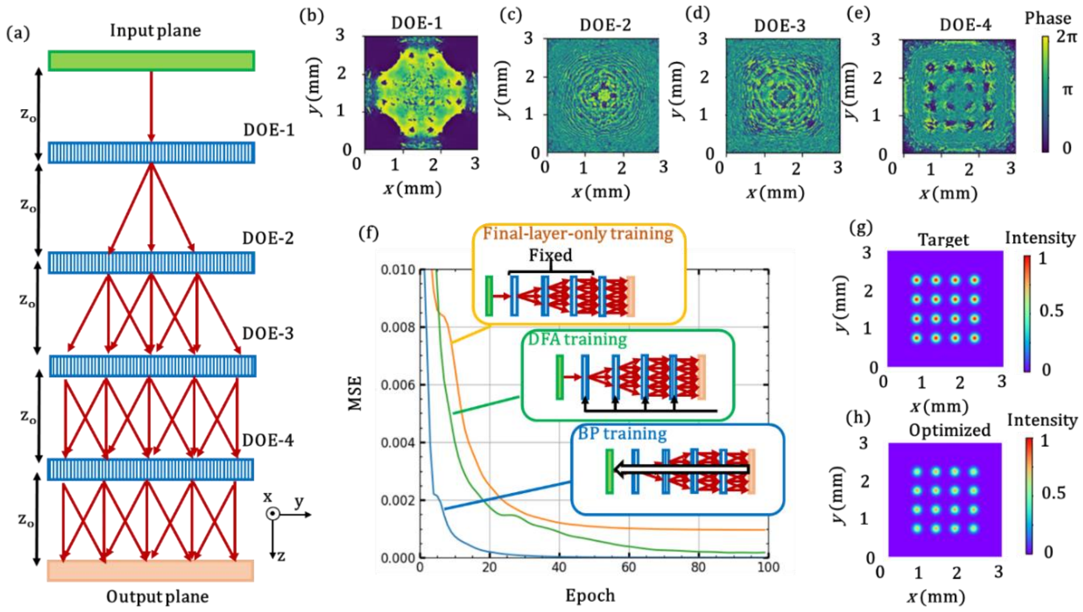

Fig. S.11. ***DFA for diffractive deep neural network.*** *(a) Schematic illustration of deep diffractive neural network composed of diffractive optical elements (DOEs). Optimized phase pattern for (b) first, (c) second, (d) third, and (e) fourth layer. (f) Numerical experimental mean square error (MSE) as a function of the number of network layers. (g) Target and (h) optimized optical intensity on the output plane. Data in this figure were obtained using standard CPU/GPU computation.*

modulate the optical wavefront by tuning local refractive index distributions. In this framework, we consider the complex-valued optical amplitude on the DOE plane as the complex-valued neuron response. The optical beam propagation within the D^2NN can be described by using the same formula as that for fully connected complex-valued neural networks. The neuron weight corresponds to the complex transmission coefficient $T^{(l)}(x,y)$ of the DOEs on the lth DOE plane, which can be described as $T^{(l)}(x,y) = A^{(l)}(x,y)\exp\{j\Phi_i^{(l)}(x,y)\}$, where $A^{(l)}(x,y)$ and $\Phi^{(l)}(x,y)$ are the modulated amplitude and phase on the lth DOE plane. For a phase-only D^2NN architecture, the amplitude $A^{(l)}(x,y)$ is assumed to be a constant value equal to 1. The D^2NN can be applied to not only the machine learning accelerator demonstrated in main article and above section, but also to the inverse design of functional optics such as optical splitters and optical mode converters. Here, we numerically demonstrate the inverse design of a 1:16 optical splitter using the DFA algorithm.

In the demonstration, we assumed four-layer 3×3 mm^2 phase-only DOEs. The distance between DOEs was set to 50 mm. The input beam was assumed to be a single gaussian beam with the width of 100 μm. The target was set to 16 gaussian beams with the width of 100 μm [see Fig. S11(g)]. The grid size on the DOE plane was set to 5 μm, and the input wavelength was 1.5 μm. We assumed linear optics in this demonstration (there is no nonlinear activation). The forward propagation was calculated by the beam propagation method. For the optimization, we used the DFA algorithm. Figure S.11(b)–(e) show the designed phase patterns after the training. As can be seen, the designed pattern for each

layer differ from each other, suggesting that each layer has a different optical function. Figure S.11(f) shows the mean square error (MSE) as a function of the training epoch. For comparison, the results with the adjoint method (continuous representation of BP) and final-layer-only training are also plotted. As can be seen, the MSE of both BP and DFA training was lower than that for the final-layer-only training. This suggests successful training in the former layer. Although the MSE for the model trained by DFA is slightly inferior to that for BP, the optimized beam pattern well agrees with the target pattern. As described in the main article, DFA incurs less computational cost and is highly robust to estimation error in a physical system. Thus, it may be useful for the fast and robust inverse design of physical systems, including D^2NNs. Further investigation remains as future work.

Supplementary note S3. Possible implementation of deep RC based on delay-based dynamics

Possible architectures for deep RC using delay-line-based implementations are listed in Fig. S12. The simplest approach is spatially stacked deep RC, which is illustrated in Fig. S12(a). In this architecture, the multiple photonic RC layers are cascaded layer by layer. This architecture can support real-time processing, and the reservoir parameters (e. g. feedback gain, mask function, output weight) can be tuned independently. However, physical architectures become complex because they require many optical components. Another approach is wavelength-division deep RC [Fig. S12(b)]. In this scheme, each wavelength corresponds to each layer for deep RC. Since the photonic circuits can be shared in this architecture, their complexity is reduced compared to spatially stacked deep RC. However, we still need multiple transmitters and receivers to implement multiple layers. The other approach is the time-sharing deep RC shown in Fig. S12(c). In this scheme, the optics is exactly the same as that in the conventional single-layer photonic RC. The memorized output signals are re-input to the RC system after the first epoch. Since all the RC layers share a single hardware component, the device architecture is simple and easy to construct.

	(a) Spatially stacked deep RC	(b) Wavelength multiplexed deep RC	(c) Time shared deep RC
	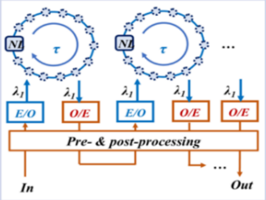	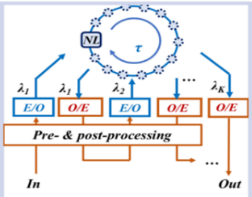	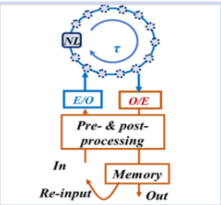
Device complexity	Bad (Need many optical components)	Acceptable (Can share optical delay line)	Good (Can share every components)
Scalability	Acceptable (Limited by device complexity)	Good	Acceptable (Limited by device complexity)
Parameter tunability	Good (Can tune all parameters independently)	Acceptable (Need to share delay line characteristic)	Acceptable (Need to share delay line characteristic)

Fig. S12. ***Possible photonic implementations of deep RC and their comparison.*** *Schematic of photonic implementation of deep RC using (a) space, (b) wavelength, and (c) time division multiplexing scheme.*

Supplementary note S4. Image recognition scheme

We describe the processing procedure for an image recognition task using deep RC. Figure S13 shows the processing method for $V\times H$ sized images using delay-based deep RC. The $1\times V$ sized temporal input signals $x^{(1)}(n)$ are masked by a $V\times N$ sized random input mask (N is the number of reservoir nodes), and they are input to the delay-ring-based reservoir layer. The discretized dynamic responses in the reservoir layer are considered as virtual nodes of the reservoir. The measured response is considered to be the next temporal input for the second layer, $x^{(2)}(n)$. To classify the input digit image to 10-class (0, 1, 2,..,9), 10 × 1 dimensional outputs $y(n')$ are obtained from weighted summation of virtual nodes in the final reservoir response $x^{(L)}(n)$ as described in the following form:

$$y_k(n') = \sum_{j=0}^{H-1} \sum_{i=1}^{N} \omega_{ijk} x_i^{(L)}(n-j) \qquad \text{(S3)}$$

where n' is the time step for the output series with the time step interval of H, and y_k is a kth element of output y.

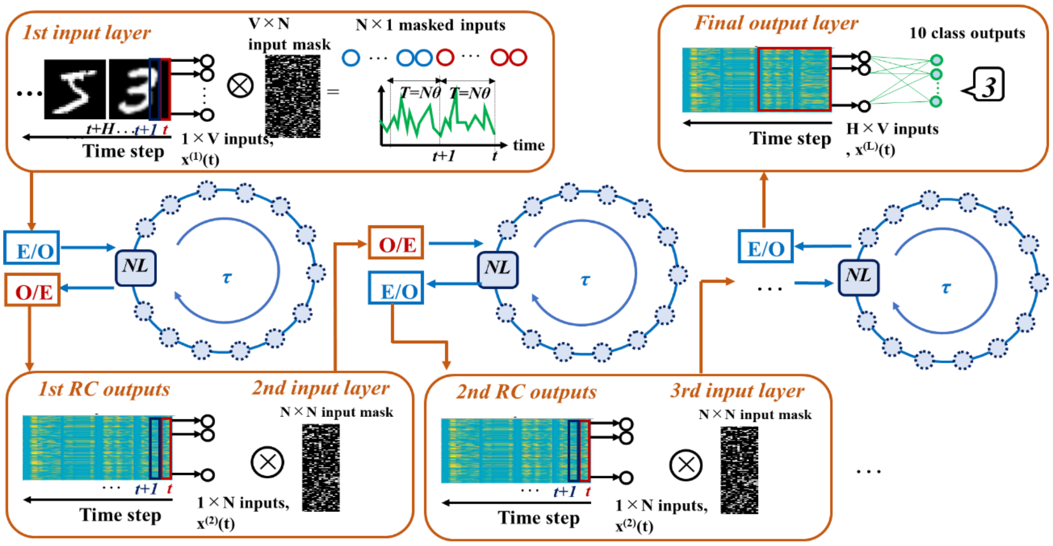

Fig. S13. ***Image processing method for delay-based deep RC.*** *For temporal processing in RC system, V×H sized input images are considered as V×1 time-series. They are masked in the digital domain, and converted to optical analog signals to introduce delay-based RC system. Optical outputs from RC system are converted to digital analog signal and re-masked in the digital domain. These signals are re-input to the RC system, which can be considered as deep reservoir network. The mask parameter of each layer is trainable thanks to our augmented DFA approach.*

Supplementary note S5 Optimization of alternative nonlinearity using genetic algorithm

In this work, we introduced alternative activation *g(a)* for the training. One question is how to find the optimal *g(a)* when we do not know the shape of *f(a)*. Here, we describe an approach utilizing a

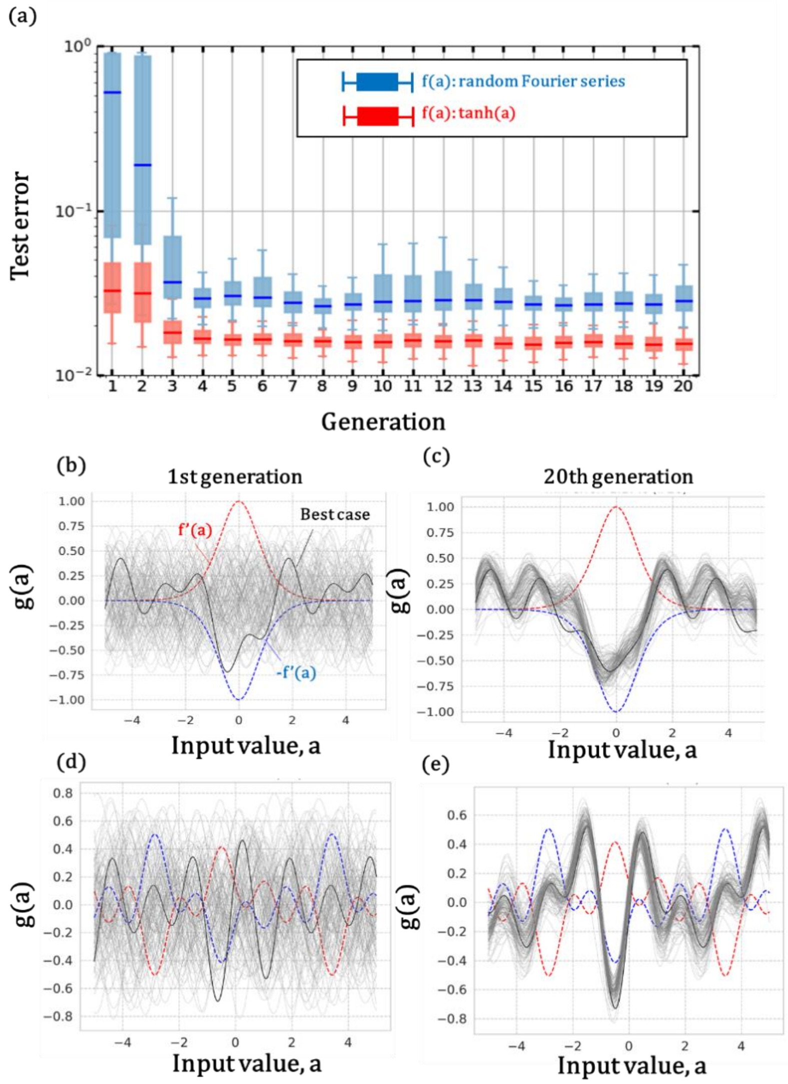

Fig. S14. ***GA-based optimization of alternative nonlinearity in augmented DFA.*** *(a) Test error as a function of generation in the PSO optimization. (b), (d) Initial g(a) shape for the case of (b) tanh and (d) random Fourier activation function. (c), (e) g(a) shape of 20th generation for the case of (b) tanh and (d) random Fourier activation function. Data in this figure were obtained using standard CPU/GPU computation.*

genetic algorithm (GA) called the particle swarm optimization (PSO) method[21]. Although it is hard to implement in a physical system, we can find a good solution for complex physical nonlinearity.

As a demonstration, we numerically examined four-layer fully connected networks with two nonlinear activation functions. One is a hyperbolic tangent (tanh). The other follows random Fourier series $f(a) = r_1 + \sum_{k=1}^{N} p_k \sin(ka) + q_k \cos(ka)$, where p_k, q_k, and r_1 are the random uniform coefficients sampled from $\mathbb{R} \in [-1:1]$ with N set to 4, and they are normalized by the relationship

$|r_1| + \sum_{k=1}^{N} |p_k| + |q_k| = 1$. The node count in each hidden layer was set to 800. For the optimization, we initialized the $g(a)$ using another Fourier series $g(a) = r'_1 + \sum_{k=1}^{N} p'_k \sin(ka) + q'_k \cos(ka)$, where p'_k, q'_k, and r'_1 are constrained the same as in the case for *f(a)*. Based on the experiment, we updated the shape of *g(a)* in the standard manner for the PSO method. For the optimization, the number of generations and number of groups were set to 20 and 128. Figure S14(a) shows the test error after the 20-epoch training as a function of the generation in the PSO algorithm. The shapes of *g(a)* for the first generation and final (20th) generation are plotted in Fig. S14(b)-(e). As can be seen in Fig. S14(a), both the average and best error decreased with the generation number, which suggests the success of our algorithm. In addition, the shape of *g(a)* converged to near the shape of $f'(a)$. Interestingly, the achieved best error of 1.14% [for *f(a)*: tanh(a)] and 1.89% [for *f(a)*: random Fourier series] were almost comparable to the *g(a)=f'*(a) case, though the converged *g(a)* shape did not agree with the shape of *f'*(a). This suggests the robustness of the proposed augmented DFA scheme.

Supplementary note S6 Augmented DFA in RC

We describe the augmented DFA for deep RC. Based on the standard BP algorithm, the gradient $\delta x^{(l)}$ in the deep RC can be described as

$$e^{(l)} = \{\Omega^{(l)} \frac{\partial x^{(l)}(n+1)}{\partial x^{(l)}(n)} + M^{(l)} e^{(l+1)}(n)\} \odot f'\{a^{(l)}(n)\}, \quad \text{(S4)}$$

where

$$a^{(l)}(n) = \Omega^{(l)} x^{(l)}(n-1) + M^{(l)} x^{(l-1)}(n). \quad \text{(S5)}$$

By neglecting the first term in eq. (S4) and replacing the second term with a linear random projection of the error signal at the final layer, $e^{(L)}(n)$, we can derive the following update rule:

$$e^{(l)} = \left[B^{(l)} e^{(L)}(n)\right] \odot f'\{a^{(l)}(n)\}. \quad \text{(S6)}$$

The almost same discussion for a recurrent neural network (RNN) with DFA training and its effectiveness have already demonstrated by Murray *et al*[22]. By augmenting the DFA algorithm to arbitrary nonlinearity *g(a)*, the same as for fully connected layers described in the main article, we finally obtain

$$e^{(l)}(n) = \left[B^{(l)} e^{(L)}(n)\right] \odot s^{(l)}(n), \quad \text{(S7)}$$

where we consider two conditions of $s^{(l)}(n)$ as follows:

$$s^{(l)}(n) = g(\Omega^{(l)} x^{(l)}(n-1) + M^{(l)} x^{(l-1)}(n)), \tag{S8}$$
$$s^{(l)}(n) = g(\Omega^{(l)} s^{(l)}(n-1) + M^{(l)} x^{(l-1)}(n)). \tag{S9}$$

From the eqs. (S7)–(S9), we can derive the update rule for each gradient for the trainable parameters as follows.

$$\delta M^{(l)}(n) = \frac{\partial E}{\partial M^{(l)}} = -e^{(l)}(n) x^{(l),T}(n), \tag{S10}$$

$$\delta \omega(n) = \frac{\partial E}{\partial \omega} = -e^{(L)}(n) x^{(L),T}(n), \tag{S11}$$

The equations (S7) and (S9)–(S11) corresponds to the eq. (8)–(11) in the main text.

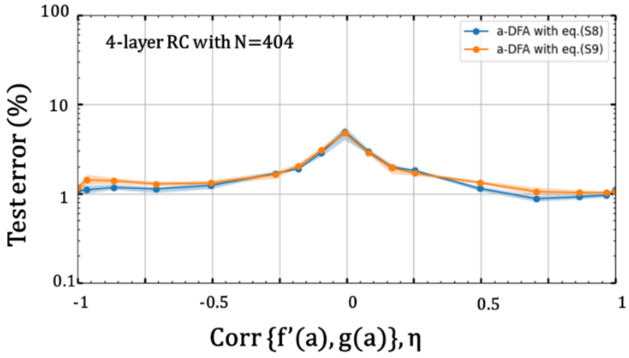

Fig. S15 ***Training procedure dependency for deep RC****. η dependency of the test accuracy of 4-layer RC model trained by augmented DFA with eq. (S8) and (S9) using MNIST dataset. The accuracy was insensitive to the choice of the equations. Data in this figure were obtained using standard CPU/GPU computation. Each experiment was repeated five times.*

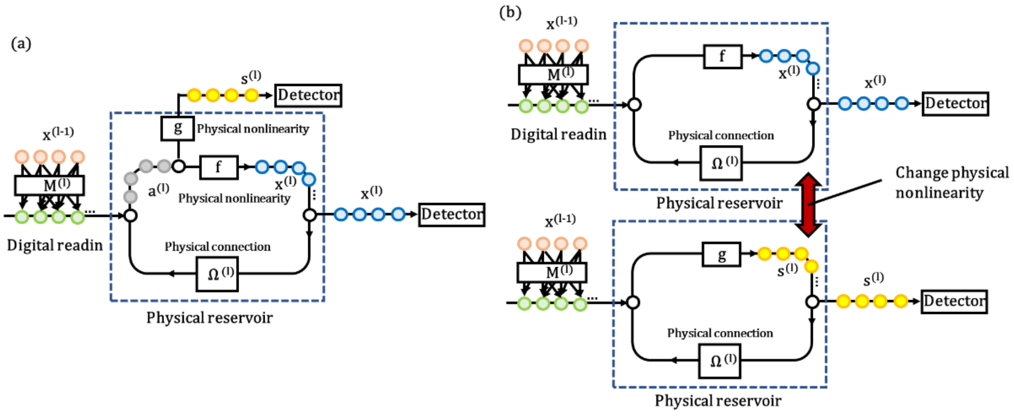

Fig. S16 ***Physical implementation of augmented DFA training for physical RC****. Schematic of possible physical*

implementation of augmented DFA training with (a) solving eq. (S8) and (b) eq. (S9). Both sets of hardware are implemented by expanding standard physical RC hardware. Thus, the device also supports the computation of forward propagation.

To check the performance difference, we compared the test accuracy of four-layer deep RC model trained by augmented DFA with eq. (S8) and (S9). Figure S15 shows the η dependency of the test accuracy for both training methods. For this numerical experiment, we used $f(a)=\cos(a)$ and $g(a)=\sin(a+\theta)$. By scanning the θ value, we swept the η value. As can be seen in the Fig. S15, the results for eq. (S8) and (S9) showed almost the same performance and trend, suggesting the performance of the augmented DFA is robust to the choice of these equations. To check the task dependence, we also compared the accuracy using MNIST, Fashion-MNIST, and CIFAR-10 datasets. The results are summarized in Table. S1. Although the performance using the training with eq. (S8) was slightly superior to that in the case of MNIST and CIFAR-10, the opposite result was observed for Fashion-MNIST. The observed difference was small for the examined case. In addition, we also found that the node count dependency of the test accuracy was almost the same (see section S12). These results suggest that both training methods are acceptable for the training of deep RC.

Next, we consider the hardware implementability of the training procedure. The implementability of the first term in the eq. (S7) [i.e. $B^{(l)}e^{(L)}(n)$] is the same as in the case for the MLP model, which is discussed in the main article and section S9. Regarding the second term in the eq. (S7) [i.e. $s^{(l)}(n)$], we have two choices of equations as described above. In principle, both the eqs. (S8) and (S9) are physically computable by expanding the standard physical RC concept. Figure S16 shows a simplified schematic of the physical RC hardware for the augmented DFA training. The architecture for solving eq. (S8) is shown in Fig. S16(a); the one for solving eq. (S9) is shown in Fig. S16(b). Although the training with eq.(S8) showed slightly superior performance to that with eq. (S9), it requires additional hardware, namely an additional nonlinear device and detector, including an analog to digital converter, as shown in Fig. 16(a). This makes the hardware and software implementation more complex. On the other hand, we can solve eq. (S8) using the same physical hardware for the forward propagation by tuning the physical nonlinearity from f to g as shown in Fig. S16(b). This implementation is easier than the other case for the optoelectronic RC system because we can easily tune the nonlinearity by controlling the bias point of the Mach-Zehnder modulator as described in the main article. Thus, in our proof-of-concept study, we used eq. (S9) for the training of our constructed physical hardware.

	**MNIST**	**Fashion MNIST**	**CIFAR-10**
**a-DFA with eq. (S8)**	99.03%	86.31%	56.85%
**a-DFA with eq. (S9)**	98.96%	86.52%	55.80%

Table S1. ***Training procedure dependence of benchmark results.*** *The best accuracy of benchmarks (MNIST, Fashion MNIST and CIFAR-10) using deep RC with 101 to 606 nodes and one to five layers. The training was executed by*

augmented DFA using eqs. (S8) [upper side] and (S9) [lower side]. The accuracies were robust to the choice of procedure.

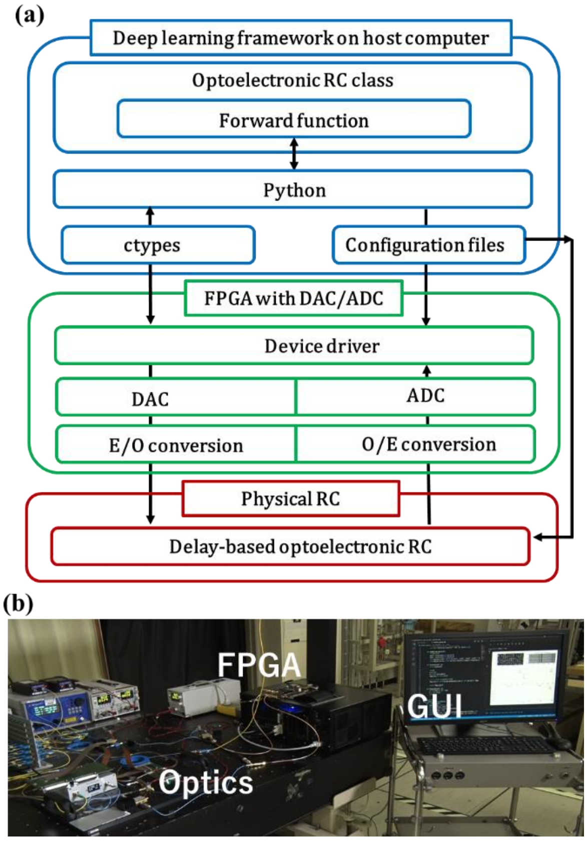

Fig. S17. ***Schematic of constructed software/hardware for optoelectric RC.*** *(a) Schematic of processing flow of constructed optoelectronic RC system, (b) Image of constructed optoelectronic benchtop.*

Supplementary note S7. Software and hardware interface for physical implementation

To execute the computation using optoelectronic-RC-based physical neural networks (PNNs), we need a functional connection between the analog-optical module and deep learning framework on a standard digital computer. Here, we developed the software/hardware interface shown in Fig. S17(a). A photograph of the constructed optoelectronic hardware for deep reservoir computing is shown in Fig. 17(b). In this interface, we implemented a new class for the optoelectronic RC on Pytorch, which can be executed like a conventional network model, such as a fully connected layer and convolution layer. On the user interface, we can name the optoelectronic RC like a standard CPU or GPU by only describing *device="oe_rc"* in the python code. When the optoelectronic RC class is called, the physical parameters (e.g. feedback gain and node count) are tuned to realize a programmed setup through the configuration files. For the forward propagation, the data is passed to the FPGA via ctypes. To reduce the latency for the data transfer, we also employed an encoder and decoder whose operation speed is INT10 at 32.8 Gops/s and 131.0 Gops/s. After that, the data were aligned in a static random

access memory (SRAM) and input to/output from the optoelectronic RC through a multichannel A/D converter and O/E converter. The obtained results correspond to the computation results of eqs. (4) and (5) in the main article. These computational results are stored in the memory of the host computer. For the training phase, we update the weights using the stored results and eqs. (8) and (9). These computations are also executed on the host computer.

Supplementary note S8. Noise dependence

For the physical implementation, the effect of noise is important because the analog physical signals (e.g., optical intensity, electric field, distortion of soft-body) are always affected by noise in the physical device (e.g., shot noise, vibration). Here, we investigated the effect of noise by considering the following noise addition.

$$f'(a) = f(a + \varepsilon\sigma), \tag{S8}$$

$$g'(a) = g(a + \varepsilon\sigma), \tag{S9}$$

where σ is white gaussian noise, and ε is noise intensity. By replacing the *f(a)* and *g(a)* values in eqs. (3), (6), and (9) in the main article with f'(a) and g'(a) described in eqs. (S8) and (S9), we numerically examined the effect of noise on the performance using the MNIST dataset. For this experiment, we employed a four-layer fully connected network and four-layer RC. *f(a)* and *g(a)* were set to cos(a) and sin(a), respectively. The other experimental conditions were the same as the ones for the numerical experiments in the main article. Figure S18(a) and (c) shows the test error as a function of ε for the fully connected network and deep RC. For the deep RC training, the results for the training with eq. (S8) [see blue plot] and (S9) [see orange plot] are plotted. The ε dependency of the alignment angle is plotted in the Fig. S18(b) and (d). As can be seen, the performance and alignment angle below $\varepsilon=10^{-1}$ are almost same as in noiseless case. In addition, the results for deep RC training with eq. S8 and S9 shows almost same performance, suggesting both training is usable for noisy deep RC training. Our optoelectronic RC benchtop showed the signal-to-noise-ratio (SNR) of 24 dB, which corresponds to $\varepsilon=4 \times 10^{-2}$. Thus, we think that the effect of gaussian noise (e.g., noise from the amplifier and photodetectors) on the physical computation is relatively small. The effect of noise has also been investigated in work on differential privacy with DFA[23,24].

Supplementary note S9. Physical implementation of linear random projection

In computing eq. (2), (3), or (8) in the main article, the linear random projection ($B^{(l)}e^{(L)}$) is the bottleneck for faster and energy-efficient computation. One of the solutions is to implement a physical

implementation of this operation. As this operation is the same as that for the FC layer without nonlinear activation and weight training, we can, based on various previous work, use a physical setup for the multi-layer-perceptron (MLP), extreme learning machine (ELM), and RC. Figure S19(a) shows one example based on a large-scale optical implementation[25–27]. We assumed an intensity modulator, such as an organic light-emitting diode (OLED) display or diffractive mirror device (DMD), for the input encoding. Thus, the input vector [$e^{(L)}$ for the processing of DFA] is reconfigurable. By using an additional SLM as the random scattering medium, we can implement programmable random matrices $B^{(l)}$. Note that $B^{(l)}$ are typically complex-valued matrices. We also assumed the intensity detection of random projected signal using a CMOS or CCD camera. Although we can measure $B^{(l)}e^{(L)}$ directly by measuring the complex-valued optical field using a wavefront camera or coherent detector, these detectors are unscalable at present. Thus, we assumed intensity measurement here. As recent SLMs have full-HD (1920 × 1080) or 4K (3840 × 2160 pixels) resolution, we can implement 10^6 to 10^7 matrix operation in principle. The 5×10^5 by 5×10^5 matrix operations on a single integrated optics have been reported experimentally[25] (note that this demonstration used a fixed diffuser as random matrix, which is not reconfigurable).

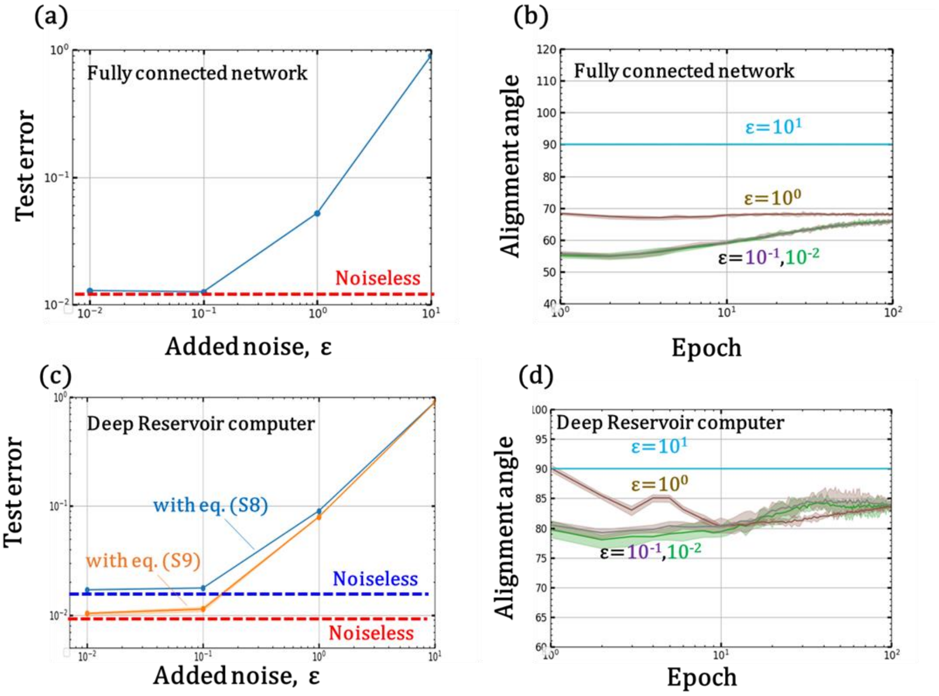

Fig. S18. ***Noise robustness of DFA.*** *(a),(c) Test error for (a) four-layer fully connected network and (c) four-layer deep RC as a function of added noise. (b),(d) Alignment angle for (b) four-layer fully connected network and (d) four-layer deep RC as a function of training epoch. Data in this figure were obtained using standard CPU/GPU computation.*

This system has a fundamental limitation compared with standard digital computing, namely a limitation due to the intensity modulation and detection. Here, we investigated the effect of this

limitation on the physical DFA training using a simple simulation setup. In the simulation, we used the four-layer multilayer perceptron (MLP) model with hyperbolic tangent (tanh) nonlinear activation, which was computed by the standard MLP model on Pytorch. For the training, we used following update rule.

$$\delta W^{(l)} = -\left[\left|B^{(l)}e^{(L)}\right| \odot g(a^{(l)})\right]x^{(l),T}. \tag{S10}$$

The difference from original one [eq. (3) in the main text] is that the intensity of random projected signal $|B^{(l+1)}e^{(L)}|$ is used in eq. (S10). Although $e^{(L)}$ is real-valued signal having both positive and negative values, the intensity modulator cannot represent a negative value. Thus, we define the alternative error signal $e'^{(L)}$ as $e'^{(L)} = e^{(L)} - \min(e^{(L)})$. To compensate for this offset, we consider the following equation by varying the eq. (S10).

$$\begin{aligned} \delta W^{(l)} &= -\left[\{\left|B^{(l)}e'^{(L)}\right| - \left|B^{(l)}min(e^{(L)})\right| \odot g(a^{(l)})\right]x^{(l),T} \quad (min(e^{(L)}) \geqq 0) \\ \delta W^{(l)} &= -\left[\{\left|B^{(l)}e'^{(L)}\right| + \left|B^{(l)}\right|min(e^{(L)})\right|\left| \odot g(a^{(l)})\right]x^{(l),T} \quad (min(e^{(L)}) < 0)' \end{aligned} \tag{S11}$$

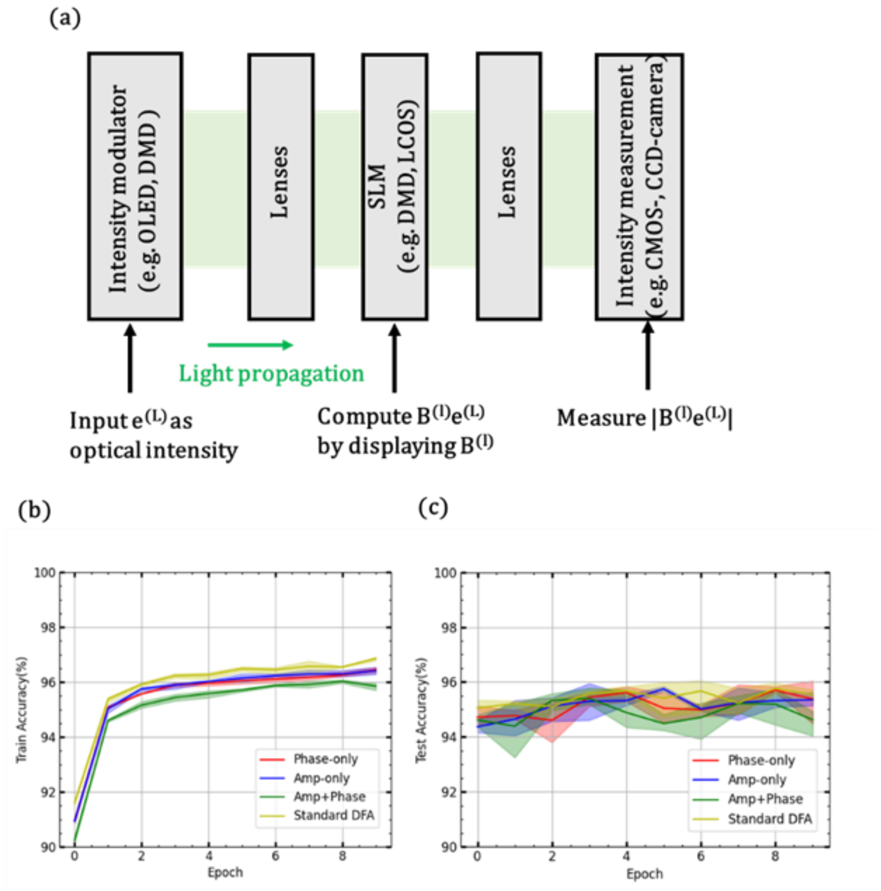

Fig. S19. ***Photonic implementation of random linear projection.*** *(a) Example of optical implementation for random linear projection. Results for (b) training and (c) test phase for MNIST benchmark using phase-only, amplitude-only,*

and both amplitude- and phase-modulated random matrix for DFA training. Data in this figure were obtained using standard CPU/GPU computation.

Both $e'^{(L)}$ and $|min(e^{(L)})|$ in eq. (S11) can be represented by intensity modulation. Also, both $|B^{(l)}e'^{(L)}|$ and $|B^{(l)}min(e'^{(L)})|$, which require the most of the computational resource in the operation, can be solved by the photonic hardware described above. The drawback is the operation has to be performed twice for each update to obtain both $|B^{(l)}e'^{(L)}|$ and $|B^{(l)}min(e'^{(L)})|$. This treatment is the same as the intensity modulated photonic-tensor-core in previous studies[28,29]. In addition, $B^{(l)}$ is typically expressed as complex-valued matrix in the photonic implementation. Thus, we compared the effect of the type of implementation of $B^{(l)}$.

In the simulation, we used the MNIST dataset as the benchmark, and we set $g = f'$ to isolate the effect of g. Each node number in the hidden layer was set to 100. We compared the results with the those for the random matrices with amplitude-only ($B_{amp}^{(l)}$), phase-only ($B_{phase}^{(l)}$) and both amplitude and phase ($B_{amp+phase}^{(l)}$) modulation. The accuracy of the training and test is shown in Fig. S19(b) and (c). As can be seen in figure, the training based on eq. (S11) worked well for the all the situations. Also, both amplitude-only and phase-only modulation worked as a random matrix in the experimented condition. The achievable accuracy is almost the same that with standard DFA. Thus, we think that we can implement scalable random linear projection in the optical system described in Fig. S19(a).

Supplementary note S10. DFA in RC and ELM

Let us discuss the effectiveness of the augmented DFA against a randomly fixed deep network[30–32] towards the application to a physical RC and ELM. For this purpose, we investigated the performance for the a deeply connected ELM and RC using the MNIST task.

Figure S20(a) shows the test accuracy as a function of training epoch for the deep neural network including randomly fixed fully connected layers (deep ELM). In this experiment, we compared the performance for the three-layer network trained by using BP and DFA. The *f(a)* and *g(a)* were set to cos(a) and sin(a) to investigate the applicability of the standard DFA algorithm at first. For the baseline, we also show the results for only-readout training. As can be seen in the figure, we could train such a network by using DFA with almost the same accuracy as the one trained by BP, both of which were superior to the one for readout-only training.

Figure S20(b) shows the test accuracy for the network including randomly fixed recurrent layers (deep RC). The standard RC comprised an input layer (fixed fully connected unit), reservoir layer (fixed recurrent unit), and output layer (trainable fully connected unit). When we train all layers in the RC, it becomes a standard RNN unit. However, in our experiment, we only trained the input and output layers (and inter-reservoir connection in the deep RC case), and the reservoir layer was fixed. Thus, we still refer to this network as an RC. The reasons we used such an experimental setup are as follows. In the physical RC framework, only the reservoir layer is typically implemented using a physical

system like in our paper. As the input layer (and inter-reservoir connection in the deep RC case) is typically implemented on a digital pre-processor, we can train these parameters to improve the performance of a physical RC in principle. However, BP is difficult to apply for such training because BP in a physical system requires precise knowledge about the system and computational simulation with large computational costs. Thus, we adopted our framework (augmented DFA) for this system. In the experiment in Fig. S20(b), we compared the performance for the single-layer and two-layer RC with DFA training. The $f(a)$ and $g(a)$ were set to $\cos(a)$ and $\sin(a)$ to investigate the applicability of the standard DFA algorithm. For the baseline, we also examined the single-layer RC with readout-only training (the detailed image-processing scheme for the deep RC is described in detail in Supplemental Material S4). As can be seen in the figure, we succeeded in training both the single-layer and stacked RC using DFA, suggesting that DFA-based training is also effective in a deep neural network including passive layers. Importantly, whereas BP requires information about the passive layer, augmented DFA does not require it because we only need the random projection of the final error. Thus, it is highly suitable for black-box passive physical networks.

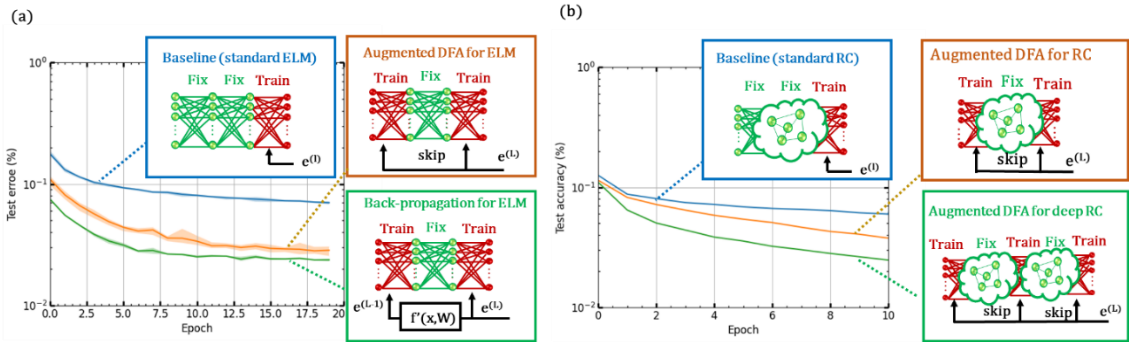

Fig. S20. ***DFA for deep RC.*** *Test accuracy for the (a) fully connected three-layer neural network with fixed frozen layer (multilayer ELM) and (b) for the one- and two-layer RC. As a baseline, the results for the standard ELM and RC approaches are also plotted in both figures. Data in this figure were obtained using standard CPU/GPU computation.*

Supplementary note S11. Alignment angle analysis

One index for evaluating whether the feedback alignment algorithm operates well or not is the alignment angle ($\angle\delta_{BP/DFA}$), which is the angle between δ_{BP} and δ_{DFA}, where δ_{BP}, δ_{DFA}, and $\angle\delta_{BP/DFA}$ are defined as $\delta_{BP} = W^T e^{(l)}$, $\delta_{DFA} = B^{(l)} e^{(L)}$, and $\angle\delta_{BP/DFA} = \cos^{-1}(\delta_{BP} \cdot \delta_{DFA})$[33]. When the alignment angle lies within 90°, the network trained by augmented DFA is roughly in the same direction as one trained by BP would be. Here, we analyzed the alignment angle of the network with four hidden layers by varying θ under the conditions $f(a)=\cos(a)$ and $g(a)=\sin(a+\theta)$ to scan η. Figure S21(a) shows the averaged alignment angle under the training as a function of η. For comparison, we also show the

results for BP by replacing $f'(a)$ with $g(a)$ in eq. (1). The evolutions of the alignment angle under the training are shown in Fig. S21(b) and (c). As can be seen, the alignment angles for the BP are significantly increased when the η is apart from one. The alignment angle is beyond 90°, which reflects the test error increase shown in Fig. 2(c) in the main article. On the other hand, the alignment angle for augmented DFA is highly robust to the η value and smaller than 90°. These results suggest that we can train a deep physical network using inaccurate $f'(a)$ (or even using an alternative nonlinear function), which provides ease of physical implementations.

Next, we analyzed the alignment angle of the deep RC network with four hidden layers by varying θ under the conditions $f(a)=\cos(a)$ and $g(a)=\sin(a+\theta)$ to scan η. The augmented DFA training was executed by using eq. (S8). Figure S22(a) and (b) shows the average alignment angle as a function of η. We also plotted the results for the same network trained by BP for comparison by replacing f'(a) to $g(a)$. The evolutions of the alignment angle under the training are shown in Fig. S22(c)-(e). As can be seen, the alignment angle for η=0 was larger than 90°; we should avoid this region to achieve better performance. However, even in the region beyond the alignment angle of 90°, we could obtain error of around 5%. We think that the network only trains the final layer weights in this case. In our experiment, the multilayer network, like the standard RC, did not have nonlinear activation in the final layer. As the gradient in the final layer was the same as in the standard network, the weight in the final layer was simply varied to minimize the final error even in the region with $\eta = 0$. In fact, we could obtain the error of ~6% in the read-out-only training, which supports our inference. It is also noticeable that the difference of alignment angle between eq. (S8) and (S9) are small. It suggests that both training method is effective for the deep RC training. The applications of augmented DFA for other network models, including a deep ELM, MLP-Mixer, vision Transformer, and ResNet, are summarized in Supplemental Material S1.

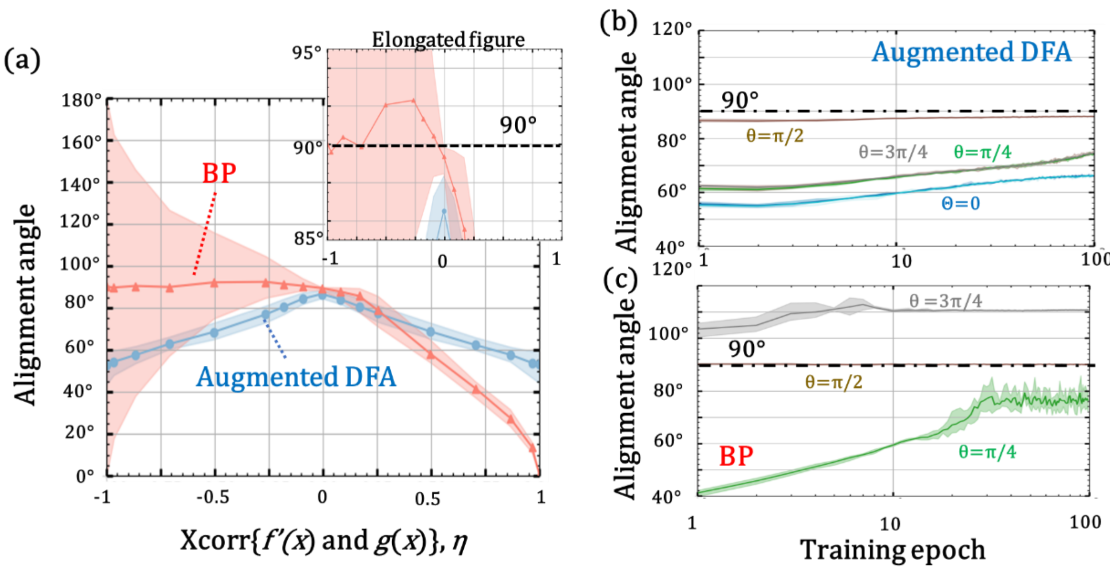

Fig. S21 ***Alignment angle in MLP.*** *(a) Averaged alignment angle for four-layer MLP model under the training as a function of η. The colored region shows the range within the minimum and maximum values. Evolution of alignment angle for layer two under training for (b) augmented DFA and (c) BP. Each experiment was repeated five times. Data in this figure were obtained using standard CPU/GPU computation.*

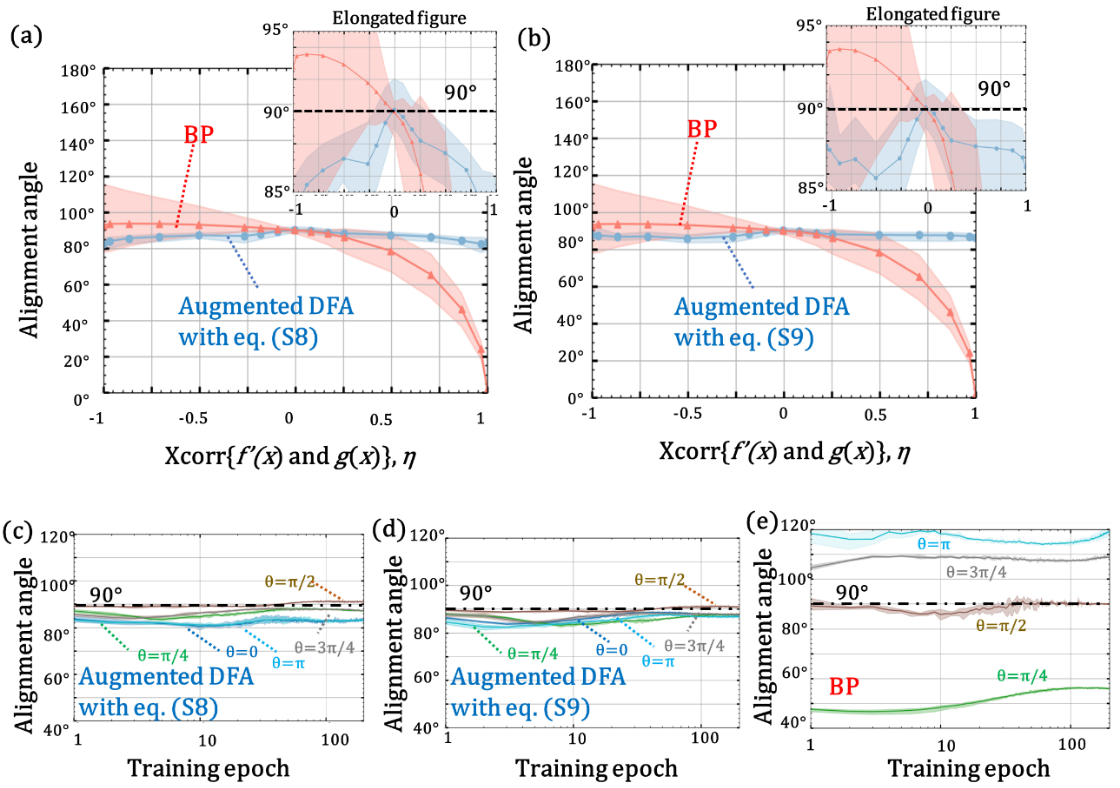

Fig. S22 ***Alignment angle in deep RC.*** *Averaged alignment angle for four-layer RC model after the training as a function of η for augmented DFA training with (a) eq. (S8) and (b) eq. (S9). The colored region shows the range within the minimum and maximum values. Evolution of alignment angles for layer two under training for augmented DFA with (c) eq. (S8), (d) eq. (S9) and (e) BP. Each experiment was repeated five times.*

Supplementary note S.12 Node dependency of robustness for augmented DFA

As described in the main article and S1–11, the augmented DFA approach is effective for various network models including a physical RC. Here, we investigated the impact of node count (N) against the robustness of the augmented DFA. In the experiment, we used a deep RC with $f(a)$=cos(a) and $g(a)$=sin(a+θ). We scanned η by sweeping the θ value. Figure S23 shows the test error for the four-layer RC model with N=101 to 808 as a function of η. Both the results of the training with eqs. (S8) and (S9) were plotted to compare the performance against the training procedure. As can be seen in the figures, the error at η=1 (i.e. the case for $g(a)$=$f'(a)$) almost saturated at N=404, suggesting that increasing the node count is not effective in this case. However, the η dependence became gentle with the node number increment. This suggests that the increment of the node is effective for further

improving the robustness in the augmented DFA training. It is also noticeable that the results for the training with eqs. (S8) and (S9) were almost same value. This result suggests that the performance of the augmented DFA is insensitive to the choice of these equations.

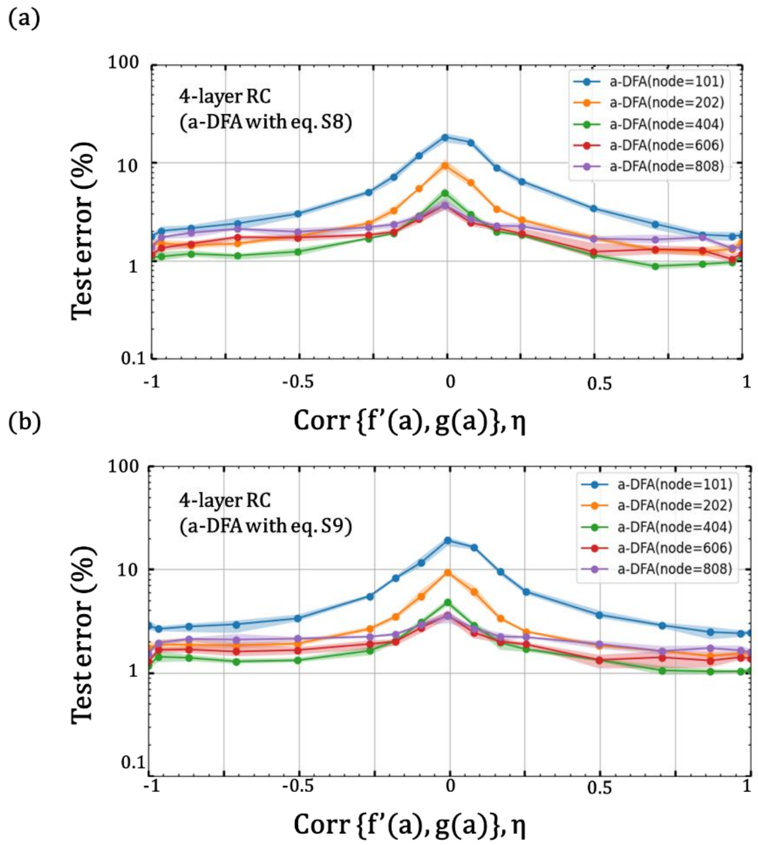

Fig. S23 ***Node count dependence of robustness with augmented DFA training.*** *Test error of four-layer RC with various node number as a function of η. η was scanned by using g(a)=sin(a+θ) by varying θ. The training was executed by augmented DFA with (a) eq. (S8) and (b) S9. Data in this figure were obtained using standard CPU/GPU computation. Each experiment was repeated five times.*

Supplementary note S.13. Spectral radius dependency of deep RC

It has been reported that the performance of an RC depends on the spectral radius of reservoir connection Ω, which corresponds to feedback gain α in the optoelectronic RC system. Here, we investigated the performance of the deep RC with 404 hidden nodes by varying the layer number and spectral radius. In the experiment, we used a deep RC with $f(a)$=cos(a) and $g(a)$=sin(a), which means that g was equal to f' in this experiment. The results for MNIST benchmark are plotted in Fig. S24. As can be seen, the augmented DFA training is effective for the whole spectral radius region. The test error of the trained deep RC depends on the spectral radius, and the best performance was obtained at the region near α=1. This region is called as "edge of chaos", and it is known that the memory capacity

(MC) of an RC system tends to be maximized in this region. Thus, the results imply that the improved performance might be due to the increment of MC near α=1. Regarding to the physical implementation, an RC system with large α value (α>1) tends to become unstable. Thus, we set the α value for the other experiment to 0.9 by considering the result in Fig. S24 and system stability.

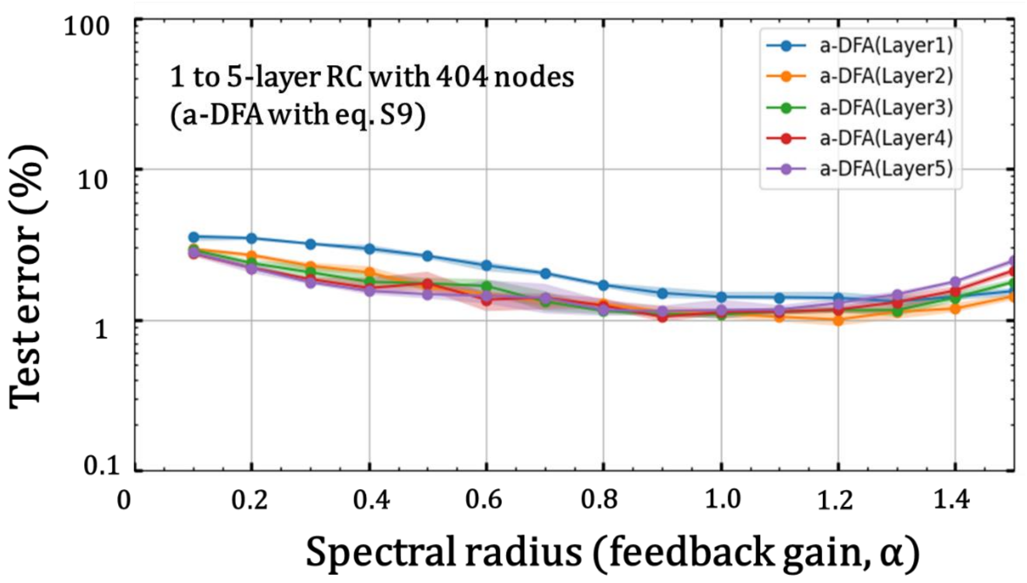

Fig. S24 ***Spectral radius dependency of augmented DFA training****. Test error for deep RC with 404 nodes as a function of spectral radius. The training was executed by augmented DFA with (S9). Data in this figure were obtained using standard CPU/GPU computation. Each experiment was repeated five times.*

Supplementary note S14. Experimental parameters

Hyper parameters for all the experiments.

A. MLP experiments (simulation in Fig. 2(a)-(c); alignment analysis in Fig. S21)

Experimental Objective: To check basic performance of augmented DFA

Benchmark: MNIST

Pre-processing: N/A

Data augmentation: N/A

Network depth: 4

Hidden node size: 800 [Fig.2(a)-(c)], and 404 (Fig. S21) for each MLP layer

Training method: BP, BP with f'(a) replaced with g(a), and augmented DFA

Cost function: Cross-entropy loss

Optimizer: Adam with learning rate of 0.0001

Initialization of random matrix B: random uniform distribution

f(a): tanh(a), cos(a)

g(a): tanh(a), sin(a), cos(a), Fourier series, triangle function

B. RC experiments

(Simulations in Fig. 2(d) and Table I; alignment analysis in Fig. S22; training method dependence in Fig. S15 and Table S1; Noise robustness in Fig. S18(c) and (d); node and layer dependency in Figs. S20(b), S23; spectral radius dependency in Fig. S24)

Experimental Objective: To investigate applicability of augmented DFA to deep RC

Benchmark: MNIST

Pre-processing: N/A

Data augmentation: N/A

Network depth: 4 (Figs. 2(d), S15, S18(c,d) and S22), 1–5 (Figs. S23 and S24, Table I and SI), 1–2 [Fig. 20(b)]

Hidden node size: 404 (Figs. 2(d), S18(c,d) and S22), 200 (for Fig. S22), and 101-808 (Figs. 2(d), S23, and S24, Tables I, and SI) for each RC layer

Training method: BP, BP with f'(a) replaced with g(a), and augmented DFA

s(*n*): solved by eq. (S9) in Figs. 2(d), S20(b), S24, and Table SI;

solved by both eqs. (S8) and (S9) in Figs. S15, S18(c,d), S22 and S23 and Table SI;

spectral radius α of 0.9 (Figs. 2(d), S15, S18(c,d), S20(b), S22-S24, and Table I and SI) and 0 to 1.5 (Fig. S24)

Cost function: Cross-entropy loss

Optimizer: Adam with learning rate of 0.0001

Initialization of random matrix B: random uniform distribution

f(a): cos(*a*)

g(a): sin($a+\theta$) where θ = 0,15,30,45,60,75,80,85,90,95,100,105,120,135,150,165,180° (Fig. 2(d), S15, and S22), and θ = 0 (Figs. S20, S23, and S24, Table I and SI)

C. Physical RC experiments (physical experiments in Fig. 4)

Experimental Objective: To check applicability to physical RC

Benchmark: MNIST, Fashion-MNIST, CIFAR-10

Pre-processing: N/A

Data augmentation: N/A

Network depth: 1—5 (for MNIST); 4 (for Fashion MNIST and CIFAR-10)

Hidden node size: 606 for each RC layer (Benchmark test); 101 to 29088 (for speed comparison)

Training method: augmented DFA, BP

Cost function: Cross-entropy loss

Optimizer: Adam with learning rate of 0.0001

Initialization of random matrix B: random uniform distribution

f(a): cos(*a*)

g(a): sin($a+\theta$) where $\theta = k\pi/10$ with k=[0,0.1,0.2,…,1]

D. Preliminary investigation for MLP-mixer (simulation in Fig. S1)

Experimental Objective: Preliminary investigation to apply DFA to MLP-mixer

Benchmark: MNIST

Pre-processing: N/A

Data augmentation: N/A

Network depth: 4 [see Fig. S1(a) and (b)]

Hidden node size: 800 for each MLP layer

Training method: BP, DFA, augmented DFA , and final-layer-only training

Cost function: Cross-entropy loss

Optimizer: Adam with learning rate of 0.001

Initialization of random matrix B: random Gaussian distribution

f(a): relu(a)

g(a): sin(a)

E. MLP-mixer (simulation in Fig. S3)

Experimental Objective: To investigate applicability to MLP-Mixer

Benchmark: MNIST, CIFAR-10

Pre-processing: N/A

Data augmentation: N/A for MNIST; random crop and random flip for CIFAR-10

Network depth: 3-mixer layer + 1 × FC layer (see Fig. S2)

Hidden node size: 4 × patch (and channel) size for each MLP (i.e. extension factor = 4)

Training method: BP, DFA (with and w/o DFL), augmented DFA (with and w/o DFL), and final-layer-only training

Cost function: Cross-entropy loss

Optimizer: Adam with learning rate of 0.0001

Initialization of random matrix B: random Gaussian distribution

f(a): relu(a)

g(a): sin(a)

F. ResNet [simulation in Fig. S5 and S6(b), (c)]

Experimental Objective: To investigate applicability to ResNet

Benchmark: MNIST, CIFAR-10

Pre-processing: N/A

Data augmentation: None for MNIST; random crop and random flip for CIFAR-10

Network depth: 17 × convolution + 1 × FC layer [see Figs. S4 and S6(a)]

Hidden node size: 3 × 3 convolution; filter size is dependent on the layer [see Figs. S4 and S6(a)]

Training method: BP, DFA, augmented DFA , final-layer-only training, BP+DFA, BP + augmented DFA

Cost function: Cross-entropy loss

Optimizer: Adam with learning rate of 0.0001

Initialization of random matrix B: random Gaussian distribution

f(a): relu(a)

g(a): sin(a)

G. ViT (simulation in Fig. S7)

Experimental Objective: To investigate applicability to ViT

Benchmark: MNIST, CIFAR-10

Pre-processing: NoneData augmentation: None for MNIST; random crop and random flip for CIFAR-10

Network depth: 3 × transformer + 1 × FC layer [see Figs. S7(a)]

Training method: BP+DFA, BP + augmented DFA , and final-layer-only training,

Cost function: Cross-entropy loss

Optimizer: Adam with learning rate of 0.0001

Initialization of random matrix B: random Gaussian distribution

f(a): relu(a)

g(a): sin(a)

H. **SNN (simulation in Fig. S9)**

Experimental Objective: To investigate applicability to SNN

Benchmark: MNIST

Pre-processing: N/A

Data augmentation: N/A

Network depth: 3

Hidden node size: 1000

Training method: augmented DFA with intensity-only modulation and measurement

Cost function: Cross-entropy loss

Optimizer: Simple gradient descent with learning rate 1.0 (no momentum)

Initialization of random matrix B: random uniform distribution

f(a): based on LIF models (See Fig. S8)

Approximated function:

$$g(a) = \mathrm{sech}^2(0.0661a) \quad (if \;\; a > 0), 0 \;\; (otherwise)$$

Derivative function:

$$g(a) = f'(a) = \frac{h_{th} t_{ref} \tau}{a(a-h_{th})\left(t_{ref}+\tau \log\frac{a}{a-h_{th}}\right)^2} \quad (if \;\; a > h_{th}), 0 \;\; (otherwise) \;\; ,$$

where

$$h_{th} = 0.4, t_{ref} = 1.0, \tau = 20$$

Optical function

$$g(a) = \cos^2(\omega a + \theta)$$

with ω=0.05, 0.1

and θ=0°, 15°, 30°, 45°60°, 75°, 90°, 105°, 120°, 135°, 150°, 165°, 180°

I. Nanophotonic neural network (simulations in Fig. S10)

Experimental Objective: To check applicability of DFA to nanophotonic deep learning

Benchmark: MNIST

Pre-processing: Downsize MNIST images from 28 × 28 to 8 × 8 size

Data augmentation: N/A

Network depth: 1–5

Hidden node size: 64

Training method: DFA, BP

Cost function: Cross-entropy loss

Optimizer: Adam with learning rate of 0.0001

Initialization of random matrix B: random uniform distribution

f(a): tanh(a)

g(a): cos (a)

J. Diffractive neural network (simulations in Fig. S11)

Experimental Objective: To check applicability of DFA to linear diffractive optics

Benchmark: Design of 1 by 16 splitter

Pre-processing: N/A

Data augmentation: N/A

Network depth: 4 (Fix-Fix-Fix-Train; train all layers)

Hidden node size: 3×3 mm^2 with 50×50 μm^2 grid-size (i.e. 600^2 equilibrium nodes)

Training method: DFA, BP

Cost function: Mean square error loss

Optimizer: Adam with learning rate of 0.0001

Initialization of random matrix B: random uniform distribution

f(a): Linear

g(a): Linear

K. Optimization of g(a) (simulation in Fig. S14)

Experimental Objective: To optimize g(a) from unknown f(a)

PSO's parameter: $(w, c_1, c_2) = (0.9, 0.5, 0.5)$

Benchmark: MNIST

Pre-processing: N/A

Data augmentation: N/A

Network depth: 4

Hidden node size: 800 for each MLP layer

Training method: augmented DFA

Cost function: Cross-entropy loss

Optimizer: Adam with learning rate of 0.0001

Initialization of random matrix B: random uniform distribution

f(a): tanh(a), cos(a)

g(a): Fourier series (search the best parameter using genetic algorithm)

L. Noise robustness (simulation in Fig. S18)

Experimental Objective: To investigate the effect of noise

Benchmark: MNIST

Pre-processing: N/A

Data augmentation: N/A

Network depth: 4 (MLP and deep RC)

Hidden node size: 404for each MLP layer; 404for each RC layer

Training method: augmented DFA

Cost function: Cross-entropy loss

Optimizer: Adam with learning rate of 0.0001

Initialization of random matrix B: random uniform distribution

f(a): cos(a)

g(a): sin(a)

M. Photonic random linear projection (simulation in Fig. S19)

Experimental Objective: To investigate possibility of photonic random linear projection

Benchmark: MNIST

Pre-processing: N/A

Data augmentation: N/A

Network depth: 3

Hidden node size: 100 for each MLP layer

Training method: augmented DFA with intensity-only modulation and measurement

Cost function: Cross-entropy loss

Optimizer: Adam with learning rate of 0.01

f(a): cos(a)

g(a): sin(a)

Optimizer: Adam with learning rate of 0.0001

Initialization of random matrix B: random uniform distribution

f(a): cos(a)

g(a): sin(a)

N. ELM experiments (simulations in Fig. S20(a))

Experimental Objective: To check applicability to fixed random network

Benchmark: MNIST

Pre-processing: N/A

Data augmentation: N/A

Network depth: 3 (Fix-Fix-Train; Train-Fix-Train,)

Hidden node size: 800 for each MLP layer

Training method: DFA, BP

Cost function: Cross-entropy loss

Optimizer: Adam with learning rate of 0.0001

Initialization of random matrix B: random uniform distribution

f(a): cos(*a*)

g(a): sin(*a*)

Supplementary References

1. Launay, J., Lighton, I. P. & Krzakala, F. Principled Training of Neural Networks with Direct Feedback Alignment. arXiv:1906.04554 (2019).

2. Han, D. & Yoo, H.-J. Efficient Convolutional Neural Network Training with Direct Feedback Alignment. arXiv:1901.01986 (2019).

3. Feldmann, J. *et al.* Parallel convolutional processing using an integrated photonic tensor core.

Nature **589**, 52–58 (2021).

4. Xu, X. *et al.* 11 TOPS photonic convolutional accelerator for optical neural networks. *Nature* **589**, 44–51 (2021).
5. Chang, J., Sitzmann, V., Dun, X., Heidrich, W. & Wetzstein, G. Hybrid optical-electronic convolutional neural networks with optimized diffractive optics for image classification. *Scientific Reports* **8**, 1–10 (2018).
6. Tolstikhin, I. *et al.* MLP-Mixer: An all-MLP Architecture for Vision. Advances in Neural Information Processing Systems **34**, 24261—24272 (2021).
7. Launay, J., Poli, I., Boniface, F. & Krzakala, F. Direct Feedback Alignment Scales to Modern Deep Learning Tasks and Architectures. *Advances in Neural Information Processing Systems* **33**, 9346--9360 (2020).
8. Feldmann, J., Youngblood, N., Wright, C. D., Bhaskaran, H. & Pernice, W. H. P. All-optical spiking neurosynaptic networks with self-learning capabilities. *Nature* **569**, 208–214 (2019).
9. Huynh, P. K., Lakshmi Varshika, M., Paul, A., Balaji, A. & Das, A. Implementing Spiking Neural Networks on Neuromorphic Architectures: A Review. arXiv:2202.08897 (2022).
10. Samadi, A., Lillicrap, T. P. & Tweed, D. B. Deep learning with dynamic spiking neurons and fixed feedback weights. *Neural Computation* **29**, 578–602 (2017).
11. Veit, A., Wilber, M. & Belongie, S. Residual Networks Behave Like Ensembles of Relatively Shallow Networks. *Advances in Neural Information Processing Systems* 550–558 (2016) doi:10.48550/arxiv.1605.06431.
12. https://github.com/lightonai/dfa-scales-to-modern-deep-learning.
13. He, K., Zhang, X., Ren, S. & Sun, J. Deep Residual Learning for Image Recognition. *Proceedings of the IEEE conference on computer vision and pattern recognition* 770-778 (2016).
14. Dosovitskiy, A. *et al.* An Image is Worth 16x16 Words: Transformers for Image Recognition at Scale. arXiv:2010.11929 (2020).
15. Shen, Y. *et al.* Deep learning with coherent nanophotonic circuits. *Nature Photonics* **11**, 441–446 (2017).
16. Smith, A. M. *et al.* Linear programmable nanophotonic processors. *Optica,* **5**, 1623–1631 (2018).
17. Zuo, Y. *et al.* All-optical neural network with nonlinear activation functions. *Optica* **6**, 1132 (2019).
18. Lin, X. *et al.* All-optical machine learning using diffractive deep neural networks. *Science* **361**, 1004–1008 (2018).
19. Zhou, T. *et al.* In situ optical backpropagation training of diffractive optical neural networks. *Photonics Research* **8**, 940 (2020).

20. Yan, T. *et al.* Fourier-space Diffractive Deep Neural Network. *Physical Review Letters* **123**, 023901 (2019).

21. Shi, Y., & Eberhart, R. A modified particle swarm optimizer. In 1998 *IEEE international conference on evolutionary computation proceedings. IEEE world congress on computational intelligence (Cat. No. 98TH8360)* (pp. 69-73). IEEE, (1998).
22. Murray, J. M. Local online learning in recurrent networks with random feedback. *Elife* **8**, (2019).
23. Ohana, R. *et al.* Photonic Differential Privacy with Direct Feedback Alignment. *Advances in Neural Information Processing Systems* **34** 22010—22020 (2021).
24. Lee, J. & Kifer, D. Differentially Private Deep Learning with Direct Feedback Alignment. arXiv:2010.03701 (2020).
25. Rafayelyan, M., Dong, J., Tan, Y., Krzakala, F., & Gigan, S. Large-scale optical reservoir computing for spatiotemporal chaotic systems prediction. *Phys. Rev. X* **10**, 041037 (2020).
26. Wang, T. *et al.* An optical neural network using less than 1 photon per multiplication. *Nature Communications* **13**, 1–8 (2022).
27. Zhou, T. *et al.* Large-scale neuromorphic optoelectronic computing with a reconfigurable diffractive processing unit. *Nature Photonics* **15**, 367–373 (2021).
28. Miscuglio, M. & Sorger, V. J. Photonic tensor cores for machine learning. *Applied Physics Reviews* **7**, (2020).
29. Feldmann, J. Parallel convolution processing using an integrated photonic tensor core. *Nature* **589**, 52–58 (2021).
30. Gallicchio, C. & Scardapane, S. Deep Randomized Neural Networks. arXiv:2002.12287 (2021).
31. Cappelli, A., Launay, J., Meunier, L., Ohana, R. & Poli, I. ROPUST: Improving Robustness through Fine-tuning with Photonic Processors and Synthetic Gradients. arXiv:2108.04217 (2021).
32. Cappelli, A. *et al.* Adversarial Robustness by Design Through Analog Computing and Synthetic Gradients. *IEEE International Conference on Acoustics, Speech and Signal Processing (ICASSP)* 3493–3497 (2022).
33. Lillicrap, T. P., Cownden, D., Tweed, D. B. & Akerman, C. J. Random synaptic feedback weights support error backpropagation for deep learning. *Nature Communications* **7**, (2016).
